# Distinctive proteomic profiles among different regions of human carotid plaques in men and women

**DOI:** 10.1038/srep26231

**Published:** 2016-05-20

**Authors:** Wenzhao Liang, Liam J. Ward, Helen Karlsson, Stefan A. Ljunggren, Wei Li, Mats Lindahl, Xi-Ming Yuan

**Affiliations:** 1 Occupational and Environmental Medicine Center, and Department of Clinical and Experimental Medicine, Linköping University, Linköping, Sweden; 2 Department of Neurology, China-Japan Union Hospital, Jilin University, Changchun, China; 3 Division of Obstetrics and Gynaecology, and Department of Clinical and Experimental Medicine, Linköping University, Linköping, Sweden; 4 Department of Clinical and Experimental Medicine, Linköping University, Linköping, Sweden

## Abstract

The heterogeneity of atherosclerotic tissue has limited comprehension in proteomic and metabolomic analyses. To elucidate the functional implications, and differences between genders, of atherosclerotic lesion formation we investigated protein profiles from different regions of human carotid atherosclerotic arteries; internal control, fatty streak, plaque shoulder, plaque centre, and fibrous cap. Proteomic analysis was performed using 2-DE with MALDI-TOF, with validation using nLC-MS/MS. Protein mapping of 2-DE identified 52 unique proteins, including 15 previously unmapped proteins, of which 41 proteins were confirmed by nLC-MS/MS analysis. Expression levels of 18 proteins were significantly altered in plaque regions compared to the internal control region. Nine proteins showed site-specific alterations, irrespective of gender, with clear associations to extracellular matrix remodelling. Five proteins display gender-specific alterations with 2-DE, with two alterations validated by nLC-MS/MS. Gender differences in ferritin light chain and transthyretin were validated using both techniques. Validation of immunohistochemistry confirmed significantly higher levels of ferritin in plaques from male patients. Proteomic analysis of different plaque regions has reduced the effects of plaque heterogeneity, and significant differences in protein expression are determined in specific regions and between genders. These proteomes have functional implications in plaque progression and are of importance in understanding gender differences in atherosclerosis.

Atherosclerosis is a chronic inflammatory disease of the arterial wall characterised by endothelial dysfunction, the recruitment of immune cells, and dyslipidaemia. Increased levels of lipids within the blood stream result in the formation of heterogeneous atherosclerotic lesions^1^. The rupture of atherosclerotic plaques containing a large necrotic core and a thin fibrous cap followed by acute luminal thrombosis is currently considered as the most common cause of acute cardiovascular events^2^.

Atherosclerosis is a complex process and the degree and patterns of intraplaque heterogeneity are not well described. Consequently, this is often less well considered in tissue sample analysis. However, it is well documented that a high degree of heterogeneity exists in composition and morphological features within individual atherosclerotic plaques in human coronary arteries^3^. Such intraplaque heterogeneity represents a challenge for plaque imaging, correlating plaque features with cardiovascular events, and for molecular understanding of mechanisms that may be used for the development of future therapeutic options.

To reveal the mechanisms leading to plaque vulnerability and plaque rupture increasing knowledge has been gained from proteomics via profiling of human atherosclerotic samples with the discovery of novel proteins and pathways involved. Proteomics has been shown to be a powerful tool in the analysis of proteins within atherosclerotic lesions^4^. A variety of proteomics techniques have been used, from gel based studies, using two-dimensional electrophoresis (2-DE) with peptide mass fingerprinting^5^,^6^,^7^, to more complex mass spectrometry (MS) techniques, utilising liquid chromatography–tandem-MS^8^. More recent studies, related to atherosclerosis, have exploited a combination of these methods^6^,^7^,^8^,^9^. However, atherosclerotic lesions are inherently very heterogeneous with regards to; lipid content, cell component, micro-haemorrhage, calcification, and fibrosis^10^, which is largely determined by where biopsies are taken. Plaque tissue heterogeneity is therefore a major limitation in plaque analyses, as results can be biased dependent on the exact location of the sample biopsy. Protein maps of atherosclerotic plaques have previously been constructed^5^,^6^,^7^,^9^. However, in most cases these maps were constructed using tissue biopsies without a clear macroscopic definition, potentially allowing for bias due to heterogeneity. Our study setup utilised comparable human carotid atherosclerotic samples and constructed a unique sampling model with biopsies sampled from different distinct pathophysiological sites, to attempt to limit the effect of tissue heterogeneity on results. Tissue biopsies were sampled from the following predefined regions; fatty streak, plaque centre, plaque shoulder and fibrous cap, and were compared to an internal control region.

The aims of this investigation were to create a comprehensive proteomic profile from different regions of the human carotid atherosclerotic plaque, in men and women, and to establish a 2-DE reference map that could be used for comparative studies of plaque development and to find new proteins of relevance that have functional roles in different regions in human atherosclerotic plaques.

## Results

### Protein mapping of human atherosclerotic plaque

**Figure 1:**
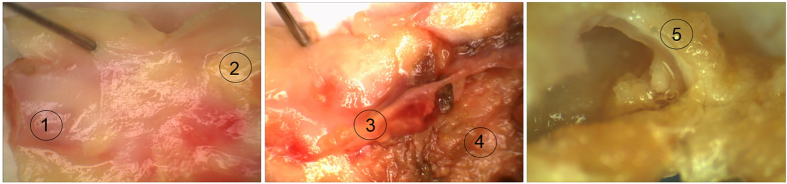
Sampling regions of carotid atherosclerotic lesion by stereomicroscopy. Carotid endarterectomy sample highlighting the different regions used for proteomic studies; 1: internal control, 2: fatty streak, 3: plaque shoulder, 4: plaque centre, 5: fibrous cap.

**Figure 2:**
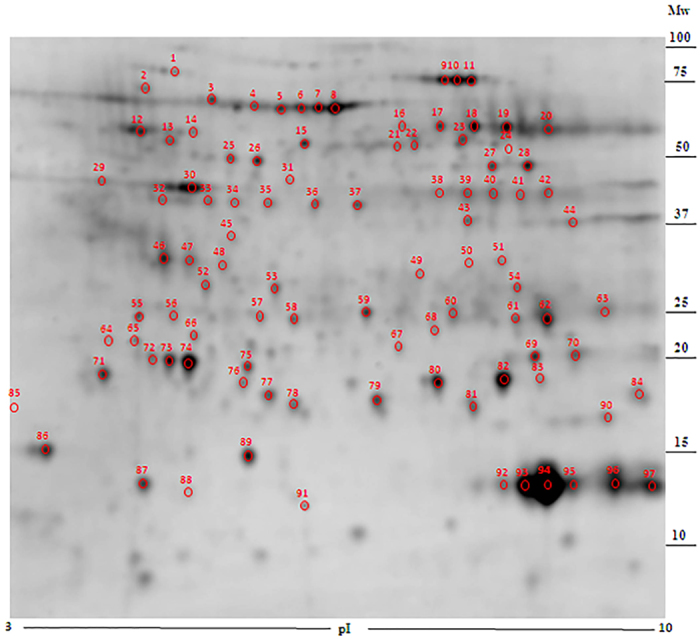
Carotid atherosclerosis 2-DE protein map. Representative 2-DE image of internal control region of carotid atherosclerotic plaque; pI 3–10 (non-linear), 300 μg of protein. Marked spots (1–97) were identified by MALDI-TOF MS analysis. (Mw; molecular weight, pI; isoelectric point).

Protein extracts from the different plaque regions; internal control, fatty streak, plaque shoulder, plaque centre, and fibrous cap (Fig. 1), were separated via 2-DE, and a reference map was determined from one of the internal control regions which contained the most number of protein spots (Fig. 2). The most abundant protein spots (n = 108) were selected for identification using MALDI-TOF MS and 97 protein spots (90%) were successfully identified, which contributed to 52 unique protein identities according to accession number (Supplementary Table S1). Secondary confirmation of protein identities was performed using tandem-MS techniques, of which 41 identities were confirmed. Compared to previous 2-DE/MS based proteomic studies on human carotid atherosclerotic lesions, 15 proteins that have not previously been identified in relation to human carotid lesions were successfully identified with the novel sampling method, of which 6 proteins were also confirmed in the tandem-MS analysis (Table 1, Supplementary Figure S1). Several proteins with the same accession number were identified in the 2-DE pattern, presumably isoforms resulting from amino acid substitutions, post-translational modifications and/or truncations.

### Site- and gender-specific alterations in carotid atherosclerotic lesions

**Figure 3:**
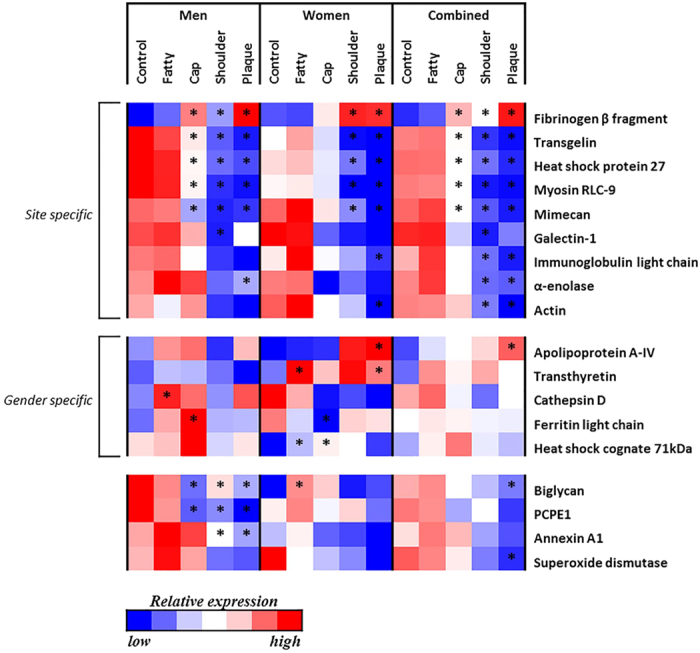
Heat map depicting differential protein expression between lesion sites per protein from 2DE/MS data analysis. Carotid endarterectomy lesions (n = 6, equal gender ratio) were sampled and defined regions isolated: internal control, fatty streak, fibrous cap, plaque shoulder and plaque centre. The colour scale represents a relative shift in protein expression between lesion sites per protein across all three categories; men, women and combined (men + women). The red colour indicates lesion sites with greater levels of protein expression, shifting through white with the median level of expression, and the blue colour indicating the lesion sites with lesser levels of protein expression. Statistical significance per protein was tested separately within each category, and each individual lesion site tested against the respective internal control values, where *p ≤ 0.05. PCPE1 - procollagen C-endopeptidase enhancer protein-1; Myosin RLC-9 - myosin regulatory light chain protein-9.

Statistical analysis of protein spot intensities identified 18 protein identities with significant differential alterations between lesion sites when compared to the internal control site (Table 2). Figure 3 displays a heat map of the relative protein expression of 18 proteins which were found to have significant differential expression. Nine of 18 proteins showed significant site-specific alterations with similar expression direction irrespective of genders (Fig. 3). Significant site-specific alterations were mainly localised to the three plaque regions (plaque shoulder, plaque centre and fibrous cap) of lesion samples with the majority of proteins displaying down-regulation, with the exception of fibrinogen β chain fragment which was up-regulated in plaque sites.

Five of the 18 proteins distinctively displayed ‘gender-specific’ trends in differential expression between lesion sites. In men a specific alteration was observed with cathepsin D expression, and in women specific alterations were observed with apolipoprotein A-IV (Apo A-IV), transthyretin and heat shock cognate 71 kDa expression. Additionally significant alterations were observed with ferritin light chain expressions in both genders. Ferritin light chain expression displayed both site- and gender-specificity. Opposing expression directions were localised to the fibrous cap site when comparing genders, up-regulated in men while down-regulated in women respectively (Fig. 3).

Additionally, significant alterations in biglycan expression were found down-regulated in men and up-regulated in women, in different lesion sites. Moreover several proteins showed similar trends in expression directions in both genders such as procollagen C-endopeptidase enhancer-1 (PCPE1), annexin A1, and superoxide dismutase (Fig. 3).

### Validation of protein alterations by tandem-MS analysis

For validation of 2-DE/MS results an additional analysis of 20 human carotid atherosclerotic lesions (internal control, fatty streak and plaque centre regions), 10 men and 10 women, was performed using tandem-MS. To retain a manageable study sample size we removed the plaque shoulder and fibrous cap regions (herein 60 protein extracts were analysed). Moreover, the plaque shoulder region was excluded as results from 2-DE/MS analysis displayed similar significant protein alterations within the plaque shoulder and plaque centre regions (Fig. 3). This resulted in the identification of over 1000 proteins (Supplementary Table S2). Of the 52 proteins identified by 2-DE mapping, 41 were also identified by sequence data from the tandem-MS analysis (Supplementary Table S1).

**Figure 4:**
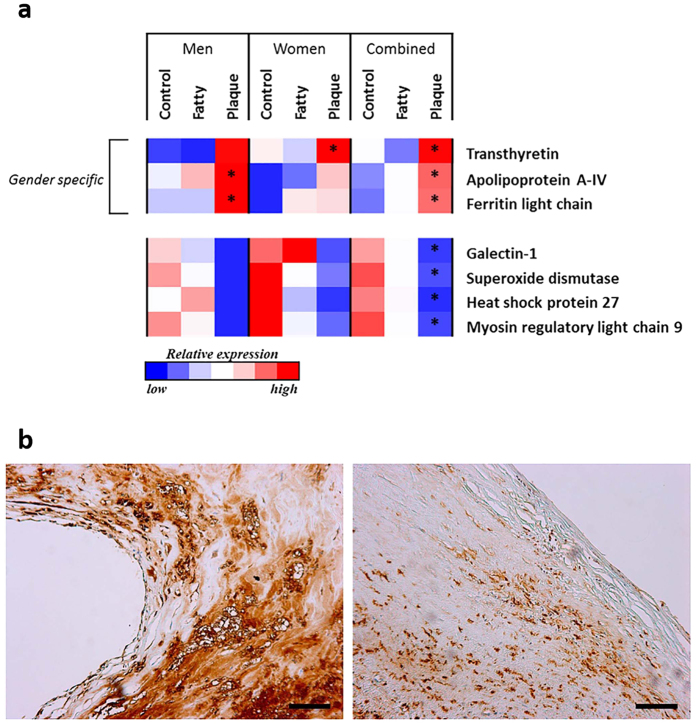
(**a**) Heat map depicting significant differential protein expression between lesion sites per protein from nLC-MS/MS data analysis. Carotid endarterectomy lesions (n = 20, equal gender ratio) were sampled and defined regions isolated: internal control, fatty streak and plaque centre. Proteins with significant alterations in expression levels determined by both nLC-MS/MS and 2-DE/MS are displayed, as means of validation. The colour scale represents a relative shift in protein expression between lesion sites per protein across all three categories; men, women and combined (men + women). The red colour indicates lesion sites with greater levels of protein expression, shifting through white with the median level of expression, and the blue colour indicating the lesion sites with lesser levels of protein expression, determined by nLC-MS/MS analysis. Statistical significance per protein was tested separately within each category, and each individual lesion site tested against the respective internal control values, where *p ≤ 0.05. (**b**) Higher levels of ferritin in male plaques is confirmed by immunohistochemistry. Representative photographs of ferritin expression in carotid plaques from male patient (left) and female patient (right). Bars = 100 μm.

In addition, statistical analysis was performed on those proteins found significantly altered from the 2-DE results. Although only three different plaque regions were analysed by tandem-MS, the results showed 7 proteins that displayed significant alterations when comparing the internal control and plaque centre regions (Fig. 4a). Significant alterations which were validated using both 2-DE and tandem-MS included the down-regulations of galectin-1, superoxide dismutase, heat shock protein 27 (Hsp27) and myosin regulatory light chain-9 (myosin RLC-9) in the plaque centre region of the gender combined analysis. Also the significant upregulation of transthyretin in the plaque centre of women was validated using both techniques.

The increased number of patients samples used in the tandem-MS analysis also confirmed trends in alterations observed from 2-DE results. From tandem-MS analysis ferritin light chain and Apo A-IV displays significant increases in the plaque centre of men (Fig. 4a). Validation of immunohistochemistry showed that levels of ferritin were profoundly increased in the plaques from male patients in comparison with plaques from female patients (Fig. 4b). Quantitative results of image analysis revealed that plaques from 36 male patients showed significantly higher levels of ferritin as compared to plaques from 23 female patients (32.1 vs. 15.3 in median value, p < 0.05).

Conversely, the significant alteration of Apo A-IV in the plaque centre of women, observed via 2-DE/MS, was not validated by tandem-MS albeit a non-significant up-regulation was still present and significant overexpression of Apo A-IV in the plaque centre remained when gender groups are combined (Fig. 4a).

## Discussion

Protein maps have previously been constructed from human atherosclerotic plaque^5^,^6^,^7^,^9^. However, in most cases these maps were constructed using tissue biopsies without a clear macroscopic definition. Protein profiling of different regions of human carotid plaques could help identify functional implications of protein profiles in the plaque progression. From the sampling model used here a reference 2-DE map was constructed, and used to depict the protein profile in five different regions of human carotid lesions. Protein mapping revealed the expression of 15 proteins that had not previously been mapped, by 2-DE/MS, in relation to human carotid atherosclerosis. Further 2-DE/MS quantification of protein expression revealed that 9 proteins display significant differential protein expression with distinctive site-specific alterations and 5 proteins with clear gender-specific expression pattern. Tandem-MS results displayed significant alterations in 4 site-specific proteins, and 3 gender-specific alterations.

Atherosclerotic vascular remodelling requires degradation of extracellular matrix (ECM), as both a function of normal physiology and a consequence of atherogenic processes. Within the vessel wall, vascular smooth muscle cells (SMCs) can significantly contribute to the cytokine-dependent inflammatory network. The deposition of ECM, increased accumulation of leucocytes and altered levels of inflammatory mediators, may constitute SMC-fostered inflammation in atherosclerosis^11^. In our study 9 proteins, including biglycan, are implicated in the remodelling of the ECM and SMC functions were found at significantly reduced levels within atherosclerotic regions including, plaque centre, plaque shoulder and fibrous cap.

Biglycan expression was decreased in all lesion regions in men. Biglycan is known to accumulate within the ECM^12^ and to colocalise with apolipoproteins increasing their retention in the ECM of atherosclerotic arteries^13^. However when comparing the differential expression of biglycan and Apo A-IV an inverse correlation is evident, with the levels of Apo A-IV increasing in plaque centre, indicating lipid deposition.

Structural proteins of the ECM such as mimecan, a proteoglycan in the same family as biglycan and actin^14^,^15^ were found at reduced levels within pathological regions. Hsp27 and transgelin have been documented to interact with actin^16^,^17^ and display similar expression profiles with decreasing expression in pathological regions. A study into the Hsp27 functionality, within vascular SMC, showed that the presence of LDL stimulates dephosphorylation of Hsp27 from actin filaments effecting actin polymerisation^17^. Transgelin, an actin binding protein, is ubiquitously expressed within SMC and has been demonstrated as an early marker of SMC differentiation^16^ and a novel repressor of matrix metalloproteinase-9^18^. In addition, myosin RLC-9, also expressed within SMC, was found at significantly reduced levels within the pathological regions across both genders. The reduced expression of myosin RLC-9 in the plaque centre was confirmed, by both 2-DE/MS and tandem-MS techniques, in the present study. Myosin RLC-9 is involved in the contraction of smooth muscle^19^, and has been reported in human atherosclerotic lesions^7^,^19^,^20^.

Stroke and myocardial infarction are catastrophic outcomes of atherosclerotic plaque rupture, resulting in thrombus formation in the vascular lumen. In formation of platelet thrombi, fibrinogen binds to its platelet integrin receptor alpha IIb beta 3 (glycoproteins IIb-IIIa complex) which is a fundamental platelet mechanism in response to plaque rupture^21^. In our study both fibrinogen β chain and fibrinogen γ chain were identified via 2-DE/MS and displayed reduced expression, albeit non-significant. However a fragment of fibrinogen β chain was identified to have significantly increased expression across both genders within the plaque shoulder, centre and fibrous cap regions, suggesting active proteolysis of fibrinogen. This can be corroborated with results from Lepedda and colleagues^22^ who identified fibrinogen fragment D at increased levels within plaque extracts. Moreover, significant reduction of alpha-enolase expression in plaque samples observed in our study may indicate dysfunction fibrinolysis in process of plaque instability.

Plaque composition and gender differences have an impact in determining cardiovascular risk. Men differ from women in manifestation of atherosclerosis and iron metabolism. In comparison with men, women naturally have greater levels of HDL^23^,^24^ and lower levels of ferritin^25^. Women typically develop cardiovascular disease around 10 years later than men but progress rapidly after the menopause. Plaque morphology differs between men and women. Women with a carotid stenosis had more stable plaques than men, independent of clinical presentation and cardiovascular risk profile^26^. Plaques from men are associated with more cellularity, more inflammatory infiltrates, and more neovascularisation^27^.

As an alternative explanation to gender difference in atherosclerosis is that accumulation of tissue iron is implicated in the progression of atherosclerosis by increased iron-catalysed oxidative injury. Intracellular iron, stored as ferritin, potentiates oxidative stress and is a key initiator of pathological remodelling of the vascular wall^28^,^29^. Previous proteomic investigations have shown an increased expression of ferritin light chain in atherosclerotic plaque^30^,^31^; results presented here are in concordance with these results with reference to men, with a significant overexpression in fibrous cap. However, in women we find a significantly decreased expression of ferritin light chain. This decrease is independent from women having a naturally lower expression of ferritin^25^, as the significant change is relative to the respective internal control sites and they were at average age 71.4 ± 1.7 (Table 3). The gender difference in ferritin light chain demonstrated by 2-DE/MS analysis is confirmed by tandem-MS analysis. Significant increases in ferritin expression in carotid atherosclerotic lesion in men is validated by immunohistochemistry. The results together suggest that ferritin light chain being responsible for storage of iron in cells and electron transfer across the ferritin protein cage may contribute to plaque formation of carotid atherosclerosis in males by modulating oxidation of lipids within the vessel wall. The process has likely associated with dysfunctional lysosomal cathepsin in human carotid atherosclerosis^32^.

Apo A-IV is a glycoprotein that is commonly associated with high-density lipoprotein (HDL), antioxidant activity and cholesterol metabolism^33^,^34^. Low levels of HDL is an indicative risk of atherosclerosis, particularly useful in risk assessment in premenopausal women where LDL has a weaker predictive value^24^. Results presented here show a significant overexpression of Apo A-IV in the plaque centre of women by 2-DE/MS analysis, and a significant overexpression in the plaque centre of men by tandem-MS analysis. However, in both methods a significant overexpression of Apo A-IV in the plaque centre is retained when gender results are combined. These results can be interpreted that there is active lipid metabolism occurring in plaque formation that may be associated with a greater risk of CVD^35^, though a specific gender difference of Apo A-IV cannot be confirmed by tandem-MS analysis with increased sample size.

The gender difference in transthyretin in human carotid atheroma demonstrated by both 2-DE/MS analysis and by tandem-MS analysis is reported here for the first time. Transthyretin is the transporter of thyroxine and retinol owing to its association with retinol-binding protein. In addition, approximately 1–2% of total plasma transthyretin is associated with HDL by binding to apolipoprotein A-I that is crucial for reverse cholesterol transport. Overexpression of Apo-AIV in atherosclerotic plaque of women demonstrated by 2-DE/MS analysis and validated overexpression of transthyretin by both methods in the plaque centre of women suggest that women may differ from men in reverse cholesterol transport in carotid plaque formation. Interestingly, the interpretation on gender differences in HDL and ferritin light chain presented here is supported by an *in vitro* study, that when iron-laden macrophages were exposed to either oxLDL or HDL the excretion of intracellular ferritin was increased or reduced, respectively^36^.

Our study was performed by sampling from a patient group with an equal gender distribution, and by analysing the results separately by gender and also combined. Due to heterogeneity within atheroma lesions and individual patient samples, an internal control region within each endarterectomy sample was used to reduce the effect of variation. Through this we could show gender specific differences and distinctive differences in protein expression between different lesion regions. This study provides a proteomic platform in which to expand on, to provide a better understanding of gender difference in atherosclerosis in a larger scale setting.

Some differences in protein expression are observed between the two methods, 2-DE/MS and tandem-MS, which in turn highlight the advantages and disadvantages of both methods. The major advantage of using 2-DE/MS is that individual protein isoform patterns can be distinguished, and if needed quantified individually. As seen with the protein mapping numerous proteins are present in multiple positions, indicating isoforms, within the 2-DE gels (Fig. 2, Supplementary Table S1). This cannot be replicated with tandem-MS techniques where multiple isoforms are not distinguishable and are quantified cumulatively. The major advantage of using tandem-MS is the high sensitivity in protein identification that is clearly illustrated within this work. For example, LC-MS/MS is not limited by the size of proteins, to the same extent as 2-DE/MS that cannot be used to separate very large proteins within a sample, and can therefore identify substantially more proteins (Supplementary Table S2). At the same time, the high number of proteins also creates challenges in respect to e.g. false positives and ion suppression that may complicate the interpretation of data retrieved from the analyses. Thus a combination of these two techniques was used, which utilise different methods to separate and resolve proteins, to provide a better opportunity to fully comprehend the proteome of the human atherosclerotic tissue used in this study.

## Conclusions

The unique sampling method of the atherosclerotic plaque has minimised the effect of heterogeneity of atheroma on efficacy of proteomic analyses. A 2-DE reference map was successfully constructed mapping 52 unique proteins, of which 15 proteins had not previously been mapped by 2-DE/MS in human carotid atherosclerotic lesions. Eighteen proteins significantly differed in expression among different lesion sites sampled. Among them, five proteins display gender-specific expression. Complementary analysis by tandem-MS identified over 1000 proteins, confirming the identities of 41 proteins, and validating the expression trends of 7 significantly altered proteins from 2-DE/MS analysis, including gender differences in ferritin light chain and transthyretin. Our study suggests that the protein profiles with distinct preferences in lesion sites are of importance for understanding their functional implication and gender difference in atherosclerosis.

## Materials and Methods

### Human carotid artery samples

Carotid plaques were obtained from patients (n = 26, equal gender ratio) undergoing carotid endarterectomy performed within the Linköping Carotid Study (Linköping University Hospital, Linköping, Sweden). Written informed consent was obtained from all patients. All experimental protocols were approved by the local ethics committee (Linköping University Hospital Ethics Committee, Linköping, Sweden), and all methods were carried out in accordance with the approved ethical guidelines. Clinical information for included patients can be found in Table 3. Patients’ ages ranged from 60 to 83 years for men and 61 to 83 for women. More than 76% of patients received statin treatment in both men and women. Several other stroke risk factors were recorded, including hypertension (defined by hypertension history and diastolic blood pressure ≥110 mm Hg), smoking (smoking >5 years) and diabetes mellitus. All patients underwent preoperative duplex ultrasound scanning of the carotid artery and had ≥50% stenosis.

Biopsies (4 mm diameter) were taken from predefined regions of the atherosclerotic lesion (Fig. 1). Carotid atherosclerotic plaques used for 2-DE experiments (n = 6, equal gender ratio), included biopsies selected from the internal control, fatty streak, plaque shoulder, plaque centre and fibrous cap regions. Carotid atherosclerotic plaques (n = 20, equal gender ratio) used for tandem-MS analysis, where biopsies were selected from internal control, fatty streak and plaque centre regions (Fig. 1). Biopsies were rapidly frozen in liquid nitrogen and crushed to a fine powder using pestle and mortar. Protein extraction using TriZol LS reagent (Life Technologies, UK) according to manufacturer guidelines. Briefly, crushed biopsy samples were homogenised in 1 mL TriZol reagent via sonication, and phase separation was performed via addition of 200 μL chloroform. RNA and DNA containing layers, upper and interphase layers, respectively, were removed leaving the protein containing lower organic phase. Protein precipitation was performed via the addition of isopropyl alcohol, incubated for 20 minutes, and centrifuged to generate a protein pellet. Resulting protein pellets were washed with ethanol (absolute) thrice and re-suspended in 500 μL urea sample solution (6 M urea, 2 M thiourea) with 5 μL PefaBloc (Sigma-Aldrich, MO, USA). Protein concentration measurements using 2D Quant-Kit (Bio-Rad Laboratories, CA, USA) were performed according to manufacturer guidelines.

### Proteomic analysis by 2-DE

Human atherosclerotic lesion biopsy extracts (n = 30; 5 biopsies per patient sample) were separated via 2-DE, in a setup previously described by Görg *et al*.^37^ a method used by our group for various tissue and cell extracts^38^,^39^,^40^. In the present study, 300 μg protein samples were reconstituted in sample solution (9 M urea, 1% DTT, 4% CHAPS, 2% Pharmalyte pH 3–10, and 1% bromophenol blue). Proteins were isoelectrically focused on immobilised pH gradient strips (pH 3–10 non-linear IPG strip, GE Healthcare) at 46 000 Vh (max 8000 V). Second dimension was performed using home cast homogenous gels (SDS-PAGE; T = 14%, C = 1, 5%) and run overnight at 40–800 V, 10 °C, 30 mA. Resulting gels were stained with SYPRO Ruby fluorescent dye (Life Technologies), according to manufacturer instructions, and visualised using VersaDoc 4000MP system (Bio-Rad Laboratories). Gel images were further evaluated by PD Quest 2-DE software v.8.0.1 (Bio-Rad Laboratories). Protein spot intensities were quantified as parts per million (ppm) of total gel density. Protein spots detected on 2-DE were excised and subjected to in-gel trypsin digestion as described previously^41^. Peptides were identified with matrix-assisted laser desorption/ionisation-time of flight (MALDI-TOF) MS (Voyager DE PRO, Applied Biosystems, Foster City, CA, USA). Matrix used was 2,5-dihydroxybenzoic acid in 70% acetonitrile/0.3% trifluoracetic acid (20 mg/mL, mixed 1:1 with tryptic peptides). Databases NCBI, Swiss-Prot, and UniProt were searched using MS-Fit as search engine (http://prospector.ucsf.edu). Search restrictions were: isoelectric point, molecular weight, human species, mass tolerance <50 ppm, methionine oxidation, cysteine modification by carbamidomethylation, N-terminal acetylation, and a maximum of 1 missed cleavage by trypsin.

### Proteomic analysis by tandem-MS

Protein identities were also confirmed by nLC-MS/MS (LTQ Orbitrap Velos Pro; Thermo Fisher Scientific, MA, USA). Protein samples (n = 60; 3 biopsies per patient sample) were reduced and alkylated, via DTT (0.25 M) and iodoacetamide (0.75 M) treatment, before running through a 3 kDa cut-off filter (Amicon Ultra 3 K device; Merck-Millipore, Germany) to concentrate and remove potential interfering substances. Protein samples were subjected to digestion with trypsin (1:25 trypsin/protein) and resulting peptide samples were dried before reconstitution in 0.1% formic acid in water. Samples were loaded at a total protein concentration of 250 ng and separated using nanoflow HPLC system (Easy-nLC; Thermo Fisher Scientific) with a 1.5 h linear increase from 2% to 40% acetonitrile in a C18 column (100 mm × 0.75 μm; Agilent Technologies, CA, USA). Spectra were processed using MaxQuant v1.5.12 (Max Planck Institute of Biochemistry, Germany) to search against the human protein database (Uniprot, downloaded 17^th^ January 2015) using 6 ppm mass tolerance for MS and 0.5 Da for MS/MS. Peptides with a false-discovery rate of less than 1% were retained and proteins with at least 2 unique peptides were considered identified.

### Plaque processing and immunohistochemistry

All operations were performed with minimal manipulation of the specimen and without opening of the arterial lumen. Carotid artery samples were collected immediately after endarterectomy. Cross-sectional segments of each plaque were embedded in paraffin and collected in series with 5 μm sections. For immunohistochemistry, sections were deparaffinised in xylene and rehydration in graded ethanol. Serial paraffin sections were exposed to rabbit anti-human ferritin (DAKO, Denmark). The immuno-reactions were visualised using the DAKO EnVision+/HRP method. Controls without primary antibodies were run for each protocol, resulting in consistently negative results. Isotype controls were tested with normal serum from the same animal or species as the primary antibody or the same immunoglobulin isotype. Quantitative image analysis of immunohistochemistry was performed as described previously^32^.

### Statistical analysis

Differences in protein expression between different regions were measured using 2-DE quantification (protein intensity in ppm of total gel density) in combination with the protein mapping results. The quantification values of proteins which were found to have multiple isoforms within the gel were combined for statistical analysis. Comparisons of protein intensities were compared per lesion site (fatty streak, plaque shoulder, plaque centre and fibrous cap) against the internal control site. Obtained data for each group was expressed as mean ± SEM. Statistical analysis of data obtained from protein intensities of 2-DE, tandem-MS, and continuous data from clinical information were determined by non-parametric Mann-Whitney *U* test. Nominal data from clinical information was examined by Chi-square test. Probability values, p ≤ 0.05, were considered statistically significant.

### Data availability

The mass spectrometry proteomics data have been deposited to the ProteomeXchange Consortium via the PRIDE^42^ partner repository with the dataset identifier PXD003930.

## Additional Information

**How to cite this article**: Liang, W. *et al*. Distinctive proteomic profiles among different regions of human carotid plaques in men and women. *Sci. Rep.* **6**, 26231; doi: 10.1038/srep26231 (2016).

## Supplementary Material

Supplementary Information

## Figures and Tables

**Table 1:** Newly mapped proteins in relation to carotid atherosclerotic plaque.

**Spot No.**	**Protein name**	**UniProt Accession No.**	**Average MOWSE score**	**pI/Mass (kDa) (theoretical)**	**Peptides matched**	**Sequence coverage**
21	Serpin B12	Q96P63	4.10E + 03	5.4/46	8	24%
23	EH domain-containing protein 4	Q9H223	3.02E + 03	6.3/61	9	16%
24	Procollagen C-endopeptidase enhancer 1*	Q15113	1.15E + 05	7.4/47	9	29%
33–36	Fibrinogen beta chain fragment	D3DP13	1.47E + 04	6.9/39	8–10	20–33%
38–42	Biglycan*	P21810	5.08E + 04	7.2/41	6–10	15–37%
49–51, 61	Rab–39	Q14964	1.20E + 03	7.6/25	4–7	24–35%
56	ADP–ribosylation factor–like protein 15	Q9NXU5	1.27E + 02	5.4/23	4	33%
60	IGK@ protein	Q6PJF2	1.29E + 04	6.1/25	6	39%
63, 84	Rab–35	Q15286	5.39E + 03	8.5/23	4–7	34–43%
64	Synaptophysin-like protein 2	Q5VXT5	6.58E + 01	5.4/30	4	16%
65–66	Hepatoma-derived growth factor*	P51858	9.68E + 02	4.7/26	4–7	25–40%
85	Calmodulin*	P62158	1.74E + 02	4.1/16	7	40%
88	SH3 domain-binding glutamic acid-rich-like protein*	O75368	3.43E + 02	5.2/12	4	40%
90	Synoviolin	E9PN88	5.77E + 03	8.9/17	5	46%
91	Protein S100-A11*	P31949	5.68E + 01	6.6/11	4	39%

^*^Protein identity confirmed by nLC-MS/MS analysis.

Following a literature review of previous studies utilising 2-DE/MS on carotid atherosclerotic lesions, it was found that 15 protein identities had not previously been reported. Spot number refers to the protein spot position in Fig. 2.

**Table 2:** Significantly altered proteins identified from carotid atherosclerotic plaque by 2DE/MS.

**Spot No.**	**Protein name**	**UniProt Accession No.**	**Average MOWSE score**	**pI/Mass (kDa) (theoretical)**	**Peptides matched**	**Sequence coverage**
3	Heat shock cognate 71kDa*	P11142	6.85E + 06	5.4/71	18	34%
24	Procollagen C-endopeptidase enhancer 1*	Q15113	1.15E + 05	7.4/47	9	29%
27–28	Alpha-enolase*	P06733	5.22E + 06	7.0/47	15–18	38–40%
30	Actin, cytoplasmic I	P60709	1.26E + 03	5.3/41	8	24%
32	Apo A-IV*	P06727	3.85E + 03	5.3/45	11	22%
33–36	Fibrinogen beta chain fragment	D3DP13	1.47E + 04	6.9/39	8–10	20–33%
38–42	Biglycan*	P21810	5.08E + 04	7.2/41	6–10	15–37%
43	Annexin A1*	P04083	3.40E + 07	6.6/38	17	53%
46–48	Mimecan*	P20774	6.31E + 03	5.5/33	5–11	14–31%
52–53	Cathepsin D*	P07339	3.45E + 04	6.1/45	13–14	30–37%
57–59, 68	Heat shock protein 27*	P04792	7.09E + 03	6.0/23	4–10	26–60%
69	Superoxide dismutase*	P04179	5.40E + 02	7.8/19	6	29%
70, 78–83	Transgelin*	Q01995	5.53E + 03	8.9/22	6–14	21–56%
71	Myosin RLC 9*	P24844	2.12E + 01	4.8/19	6	40%
76	Ferritin light chain*	P02792	2.11E + 02	5.5/20	5	31%
77	Immunoglobulin light chain	NCBI: 194173377	1.24E + 03	5.9/22	5	31%
87	Galectin-1*	P09382	2.84E + 04	5.3/14	8	62%
89	Transthyretin*	P02766	1.35E + 03	5.5/15	5	46%

^*^Protein identity confirmed by nLC-MS/MS analysis.

Matched protein spot intensities, from the 2-DE separations, were compared per lesions site (fatty streak, plaque shoulder, plaque centre and fibrous cap) against the internal control site. Eighteen protein identities displayed significant alterations, p ≤ 0.05, as determined by non-parametric Mann-Whitney *U* test. Spot number refers to the protein spot position in Fig. 2.

**Table 3:** Clinical information of patients with carotid atherosclerosis.

**Patient group**	**Men (n** = **13)**	**Women (n** = **13)**	**P value**
Age, y ± SEM	72.6 ± 1.8	71.4 ± 1.7	>0.05
Statin treatment % (n)	76.9 (10/13)	76.9 (10/13)	>0.05
Diabetes mellitus, % (n)	15.4 (2/13)	16.7 (2/12)	>0.05
Hypertension, % (n)	92.3 (12/13)	100 (12/12)	>0.05
Smoking, % (n)	8.3 (1/12)	30.8 (4/13)	>0.05
Stenosis (%)	83.6	75.6	>0.05

Basic clinical information for the men and women, whom provided carotid endarterectomy samples, included in the present study. P value denotes the statistical difference between men and women.
