# Supplementary material for: Distinctive proteomic profiles among different regions of human carotid plaques in men and women
Source: Sci Rep. 2016 May 20;6:26231. doi: 10.1038/srep26231 (PMC4873748; doi:10.1038/srep26231)
Supplement: Supplementary Information [file srep26231-s1.pdf]

Supplementary Material:

Distinctive proteomic profiles among different regions of human carotid plaques in men and women

Wenzhao Liang[a,c†], Liam J. Ward[a,b†*], Helen Karlsson[a,b], Stefan A. Ljunggren[a,b], Wei Li[b], Mats Lindahl[a], Xi-Ming Yuan[a,b*]

Supplementary Figure S1

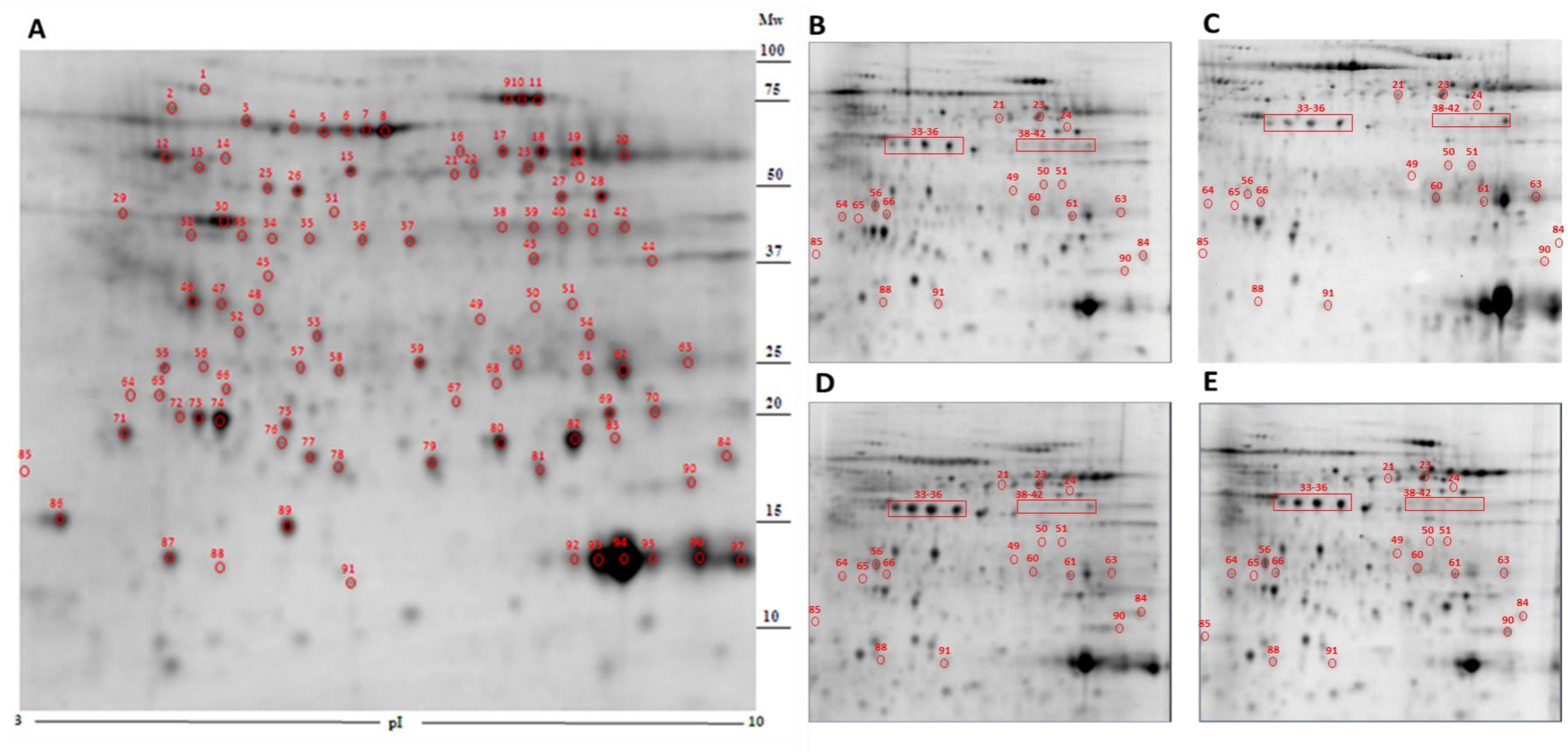

Supplementary Figure S1: Newly mapped proteins by 2-DE/MS in carotid atherosclerosis. Representative 2-DE images from the five regions sampled within human carotid endarterectomy samples; (A) internal control, (B) fatty streak, (C) plaque shoulder, (D) plaque centre, and (E) fibrous cap. Panel (A) depicts the full identification protein map which is also shown as Figure 2, panels (B-E) highlight those proteins which are newly mapped by 2-DE/MS methods in the other sampled region of atherosclerotic plaque. Newly mapped protein identities by 2-DE/MS can be found in Table 1 with corresponding spot numbers.

Supplementary Table S1

Supplementary Table S1. Identification of proteins from human carotid atherosclerotic plaque by peptide mass fingerprinting, spot no. refers to position on Figure 2. Comparison to previous studies using similar 2-DE/MS methods on atherosclerotic plaque samples was made; where previously mapped proteins have been referenced.

Spot No.	Protein name	Accession No. †	Average MOWSE score	pI / Mass (kDa) (theoretical)	Peptides matched	Sequence coverage	References
Newly mapped proteins identified by 2DE/MS in carotid plaque							
**21**	Serpin B12	Q96P63	4.10E+03	5.4 / 46	8	24%	
**23**	EH domain-containing protein 4	Q9H223	3.02E+03	6.3 / 61	9	16%	
**24**	Procollagen C-endopeptidase enhancer 1*	Q15113	1.15E+05	7.4 / 47	9	29%	
**33-36**	Fibrinogen beta chain fragment	D3DP13	1.47E+04	6.9 / 39	8-10	20-33%	
**38-42**	Biglycan*	P21810	5.08E+04	7.2 / 41	6-10	15-37%	
**49-51, 61**	Rab-39	Q14964	1.20E+03	7.6 / 25	4-7	24-35%	
**56**	ADP-ribosylation factor-like protein 15	Q9NXU5	1.27E+02	5.4 / 23	4	33%	
**60**	IGK@ protein	Q6PJF2	1.29E+04	6.1 / 25	6	39%	
**63, 84**	Rab-35	Q15286	5.39E+03	8.5 / 23	4-7	34-43%	
**64**	Synaptophysin-like protein 2	Q5VXT5	6.58E+01	5.4 / 30	4	16%	
**65-66**	Hepatoma-derived growth factor*	P51858	9.68E+02	4.7 / 26	4-7	25-40%	
**85**	Calmodulin*	P62158	1.74E+02	4.1 / 16	7	40%	
**88**	SH3 domain-binding glutamic acid-rich-like protein*	O75368	3.43E+02	5.2 / 12	4	40%	
**90**	Synoviolin	E9PN88	5.77E+03	8.9 / 17	5	46%	
**91**	Protein S100-A11*	P31949	5.68E+01	6.6 / 11	4	39%	
Previously mapped proteins identified by 2DE/MS in carotid plaque							
**1**	Alpha-1B-glycoprotein*	P04217	9.65E+02	5.6 / 55	7	15%	1
**2**	78kDa Glucose-regulated protein*	P11021	9.23E+07	5.1 / 72	18	34%	2
**3**	Heat shock cognate 71kDa*	P11142	6.85E+06	5.4 / 71	18	34%	2,3
**4-8**	Serum albumin*	P02768	1.70E+07	5.9 / 69	12-23	17-36%	3,4

**9-11**	Serotransferrin*	P02787	6.58E+12	6.8 / 77	22-36	31-50%	1
**12**	Alpha1-antitrypsin*	P01009	7.63E+05	5.4 / 47	15	47%	1,4
**13, 29**	Vimentin*	P08670	1.80E+08	5.1 / 53	23-32	45-66%	2,4,5
**14**	Heat shock protein 60*	P10809	1.19E+04	5.7 / 61	10	20%	2,3
**15**	Protein disulphide-isomerase A3*	P30101	1.81E+06	6.0 / 56	20	39%	1-3
**16-20, 22**	Fibrinogen beta chain*	P02675	1.11E+06	8.5 / 56	8-16	20-31%	1,3,4
**25-26**	Fibrinogen gamma chain*	P02679	2.01E+03	5.4 / 51	13-14	27-29%	3
**27-28**	Alpha-enolase*	P06733	5.22E+06	7.0 / 47	15-18	38-40%	1,2
**30**	Actin, cytoplasmic I	P60709	1.26E+03	5.3 / 41	8	24%	2-4
**31**	PEDF*	P36955	1.88E+04	6.0 / 46	12	28%	4
**32**	Apo A-IV*	P06727	3.85E+03	5.3 / 45	11	22%	1,3
**37**	Macrophage-capping protein*	P40121	5.02E+03	5.8 / 38	9	25%	1
**43**	Annexin A1*	P04083	3.40E+07	6.6 / 38	17	53%	2
**44**	Annexin A2*	P07355	1.96E+04	7.6 / 38	9	32%	2
**45**	CapZ-alpha1*	P52907	2.32E+02	5.4 / 33	7	23%	3
**46-48**	Mimecan*	P20774	6.31E+03	5.5 / 33	5-11	14-31%	5,6
**52-53**	Cathepsin D*	P07339	3.45E+04	6.1 / 45	13-14	30-37%	1,2
**54**	Carbonic anhydrase I*	P00915	5.66E+02	6.6 / 28	6	28%	3
**55**	Rho GDI 1*	P52565	2.16E+02	5.0 / 23	6	30%	6
**57-59, 68**	Heat shock protein 27*	P04792	7.09E+03	6.0 / 23	4-10	26-60%	1,2,5
**62, 92-94**	Haemoglobin subunit beta*	P68871	7.26E+05	6.7 / 16	7-8	49-60%	3,4
**67**	Protein DJ-1*	Q99497	3.69E+02	6.3 / 20	6	39%	3
**69**	Superoxide dismutase*	P04179	5.40E+02	7.8 / 19	6	29%	3
**70, 78-83**	Transgelin*	Q01995	5.53E+03	8.9 / 22	6-14	21-56%	4
**71**	Myosin RLC 9*	P24844	2.12E+01	4.8 / 19	6	40%	4
**72-74**	Apo A-I*	P02647	4.32E+06	5.6 / 30	7-18	30-66%	1,3
**75**	Peroxiredoxin-2*	P32119	5.75E+02	5.7 / 21	7	27%	1-3
**76**	Ferritin light chain*	P02792	2.11E+02	5.5 / 20	5	31%	1,4,5
**77**	Immunoglobulin light chain	NCBI: 194173377	1.24E+03	5.9 / 22	5	31%	4
**86**	Myosin light polypeptide 6*	P60660	1.02E+02	4.6 / 17	4	33%	4
**87**	Galectin-1*	P09382	2.84E+04	5.3 / 14	8	62%	1,2
**89**	Transthyretin*	P02766	1.35E+03	5.5 / 15	5	46%	1
**95-97**	Haemoglobin subunit alpha*	P69905	4.49E+02	8.7 / 15	4-5	20-32%	3

†Accession numbers correspond to UniProtKB database entries, unless specified

*Protein identification confirmed by nLC-MS/MS

References

1 Duran, M. C. *et al.* Atorvastatin modulates the profile of proteins released by human atherosclerotic plaques. *European journal of pharmacology* **562**, 119-129, doi:10.1016/j.ejphar.2007.01.077 (2007).

2 Viiri, L. E. *et al.* Smooth muscle cells in human atherosclerosis: Proteomic profiling reveals differences in expression of Annexin A1 and mitochondrial proteins in carotid disease. *Journal of molecular and cellular cardiology*, doi:10.1016/j.yjmcc.2012.11.002 (2012).

3 Eberini, I. *et al.* A proteomic portrait of atherosclerosis. *Journal of proteomics* **82C**, 92-112, doi:10.1016/j.jprot.2013.02.007 (2013).

4 Olson, F. J. *et al.* Consistent differences in protein distribution along the longitudinal axis in symptomatic carotid atherosclerotic plaques. *Biochemical and biophysical research communications* **401**, 574-580, doi:10.1016/j.bbrc.2010.09.103 (2010).

5 de la Cuesta, F. *et al.* A proteomic focus on the alterations occurring at the human atherosclerotic coronary intima. *Molecular & cellular proteomics : MCP* **10**, M110 003517, doi:10.1074/mcp.M110.003517 (2011).

6 Porcelli, B. *et al.* Proteomic analysis of atherosclerotic plaque. *Biomedicine & pharmacotherapy = Biomedecine & pharmacotherapie* **64**, 369-372, doi:10.1016/j.biopha.2009.10.005 (2010).

Supplementary Table S2

Supplementary Table S2. Identification list of proteins identified from human carotid atherosclerotic plaques via liquid chromatography tandem-mass spectrometry (nLC-MS/MS; LTQ Orbitrap Velos Pro, Thermo Fisher).

Protein name	UniProt accession number	Peptide matched (all)	Peptide matched (unique)	Sequence coverage [%]
1,4-alpha-glucan-branching enzyme	Q04446	5	5	17.2
14 kDa phosphohistidine phosphatase	Q9NRX4	4	4	49.6
14-3-3 protein beta/alpha	P31946	20	14	69.5
14-3-3 protein epsilon	P62258	17	14	62.4
14-3-3 protein eta	Q04917	16	13	54.9
14-3-3 protein gamma	P61981	20	15	83
14-3-3 protein theta	P27348	18	14	60.4
14-3-3 protein zeta/delta	P63104	24	20	75.9
26S protease regulatory subunit 6A	P17980	3	3	11.4
26S protease regulatory subunit 6B	P43686	2	2	12.2
26S proteasome non-ATPase regulatory subunit 1	Q99460	2	2	4
26S proteasome non-ATPase regulatory subunit 2	Q13200	5	5	11
26S proteasome non-ATPase regulatory subunit 3	O43242	3	3	7.5
28 kDa heat- and acid-stable phosphoprotein	Q13442	2	2	13.8
40S ribosomal protein S12	P25398	3	3	34.1
40S ribosomal protein S14	P62263	2	2	15.9
40S ribosomal protein S19	P39019	2	2	12.4
40S ribosomal protein S20	P60866	2	2	19.3
40S ribosomal protein S25	P62851	2	2	19.2
40S ribosomal protein S3a	P61247	2	2	4.2
40S ribosomal protein S4, X isoform	P62701	4	4	14.8
40S ribosomal protein S5	P46782	2	2	21.1
40S ribosomal protein S7	P62081	3	3	33
40S ribosomal protein SA	P08865	9	9	50.2
4F2 cell-surface antigen heavy chain	P08195	2	2	4.1
4-trimethylaminobutyraldehyde dehydrogenase	P49189	2	2	4.5
5-3 exoribonuclease 1	Q8IZH2	2	2	1.9
5-nucleotidase	P21589	7	7	22.8
60 kDa heat shock protein, mitochondrial	P10809	15	15	39.3
60S acidic ribosomal protein P0	P05388	6	6	31.2
60S acidic ribosomal protein P1	P05386	4	4	79.8
60S acidic ribosomal protein P2	P05387	4	4	59.1
60S ribosomal protein L11	P62913	2	2	14.6
60S ribosomal protein L13	P26373	2	2	8.1

60S ribosomal protein L18	Q07020	2	2	13.8
60S ribosomal protein L23	P62829	2	2	25
60S ribosomal protein L30	P62888	2	2	24.3
6-phosphofructokinase, liver type	P17858	6	5	12.2
6-phosphogluconate dehydrogenase, decarboxylating	P52209	17	17	43.3
72 kDa type IV collagenase	P08253	9	9	22.3
78 kDa glucose-regulated protein	P11021	42	42	60.6
Abhydrolase domain-containing protein 3	Q8WU67	2	2	3.2
Acid ceramidase	Q13510	9	9	32.4
Acidic leucine-rich nuclear phosphoprotein 32 family member A	P39687	2	2	18.9
Aconitate hydratase, mitochondrial	Q99798	5	5	11.3
Actin, alpha cardiac muscle 1	P68032	53	2	93.4
Actin, cytoplasmic 2	P63261	47	2	94.4
Actin, gamma-enteric smooth muscle	P63267	45	2	93.4
Actin-related protein 2	P61160	11	11	39.8
Actin-related protein 2/3 complex subunit 1B	O15143	14	13	55.9
Actin-related protein 2/3 complex subunit 2	O15144	17	17	55.3
Actin-related protein 2/3 complex subunit 3	O15145	5	5	25.3
Actin-related protein 2/3 complex subunit 4	P59998	5	5	34.5
Actin-related protein 2/3 complex subunit 5	O15511	5	5	58.3
Actin-related protein 3	P61158	16	16	61.7
Activator of 90 kDa heat shock protein ATPase homolog 1	O95433	2	2	14.2
Acyl-CoA-binding protein	P07108	5	5	65.5
Acyl-coenzyme A thioesterase 9, mitochondrial	Q9Y305	2	2	5.5
Adapter molecule crk	P46108	3	3	19.1
Adenine phosphoribosyltransferase	P07741	2	2	18.3
Adenosine monophosphate-protein transferase FICD	Q9BVA6	2	2	3.5
Adenosylhomocysteinase	P23526	5	5	14.6
Adenylate kinase isoenzyme 1	P00568	5	5	30.9
Adenylyl cyclase-associated protein 1	Q01518	30	30	64.4
Adenylyl cyclase-associated protein 2	P40123	6	6	23.3
Adipocyte enhancer-binding protein 1	Q8IUX7	39	38	42.4
Adipocyte plasma membrane-associated protein	Q9HDC9	8	8	28.1
Adipogenesis regulatory factor	Q15847	4	4	78.9
Adiponectin	Q15848	2	2	15.6
ADP/ATP translocase 3	P12236	2	2	7.4
ADP-ribosylation factor 1	P84077	7	7	54.7
Afamin	P43652	20	20	35.6
Aflatoxin B1 aldehyde reductase member 2	O43488	2	2	9.5
Aggrecan core protein	P16112	23	23	11.3
A-kinase anchor protein 12	Q02952	7	7	7.4
Alanine--tRNA ligase, cytoplasmic	P49588	2	2	4.4
Alcohol dehydrogenase [NADP(+)]	P14550	6	6	21.8

Alcohol dehydrogenase 1B	P00325	27	16	81.1
Alcohol dehydrogenase class-3	P11766	6	6	33.2
Aldehyde dehydrogenase X, mitochondrial	P30837	14	14	43.7
Aldehyde dehydrogenase, mitochondrial	P05091	14	13	46.8
Aldo-keto reductase family 1 member C1	Q04828	2	2	12.4
Aldose reductase	P15121	4	4	20.9
Allograft inflammatory factor 1	P55008	3	3	25.9
Alpha-1,3-mannosyl-glycoprotein 2-beta-N-acetylglucosaminyltransferase	P26572	2	2	3.6
Alpha-1-acid glycoprotein 1	P02763	15	9	53.2
Alpha-1-acid glycoprotein 2	P19652	14	8	54.7
Alpha-1-antichymotrypsin	P01011	30	30	61.2
Alpha-1-antitrypsin	P01009	45	45	71.1
Alpha-1B-glycoprotein	P04217	20	20	60.6
Alpha-2-antiplasmin	P08697	21	21	60.1
Alpha-2-HS-glycoprotein	P02765	17	13	53.7
Alpha-2-macroglobulin	P01023	109	101	72.3
Alpha-2-macroglobulin receptor-associated protein	P30533	3	3	7.3
Alpha-actinin-1	P12814	81	53	82.3
Alpha-actinin-4	O43707	81	52	81.3
Alpha-adducin	P35611	4	4	10.4
Alpha-centractin	P61163	4	4	22.6
Alpha-crystallin B chain	P02511	14	14	90.9
Alpha-enolase	P06733	31	28	77.9
Alpha-galactosidase A	P06280	2	2	6.1
Alpha-parvin	Q9NVD7	6	6	22.3
Alpha-soluble NSF attachment protein	P54920	4	4	18.6
Aminopeptidase B	Q9H4A4	2	2	3.4
Aminopeptidase N	P15144	22	22	33.7
Ammonium transporter Rh type A	Q02094	2	2	9.3
AMP deaminase 3	Q01432	2	2	3.4
Angiogenin	P03950	6	6	51
Angiotensinogen	P01019	14	14	32.6
Ankyrin-1	P16157	32	31	27.9
Annexin A1	P04083	30	30	74.3
Annexin A11	P50995	12	12	26.9
Annexin A2	P07355	45	45	85
Annexin A3	P12429	6	6	26
Annexin A4	P09525	21	21	66.1
Annexin A5	P08758	37	37	88.1
Annexin A6	P08133	60	60	78.6
Annexin A7	P20073	9	9	23.6
Anthrax toxin receptor 1	Q9H6X2	4	4	11
Antithrombin-III	P01008	34	34	68.5
AP-1 complex subunit beta-1	Q10567	8	3	15

AP-2 complex subunit alpha-2	O94973	7	7	15.2
AP-2 complex subunit beta	P63010	12	7	22.4
AP-2 complex subunit mu	Q96CW1	2	2	11.5
Apolipoprotein A-I	P02647	48	48	83.1
Apolipoprotein A-II	P02652	9	9	69
Apolipoprotein A-IV	P06727	41	41	80.8
Apolipoprotein B receptor	Q0VD83	2	2	2.1
Apolipoprotein B-100	P04114	375	375	77.6
Apolipoprotein C-I	P02654	6	6	37.3
Apolipoprotein C-III	P02656	3	3	30.3
Apolipoprotein D	P05090	13	13	41.3
Apolipoprotein E	P02649	40	40	83.6
Apolipoprotein L1	O14791	15	15	45
Apolipoprotein M	O95445	9	9	73.9
Apolipoprotein(a)	P08519	11	11	37.6
Aquaporin-1	P29972	7	7	48.7
ARF GTPase-activating protein GIT2	Q14161	2	2	3.4
Asparagine--tRNA ligase, cytoplasmic	O43776	2	2	2.9
Aspartyl/asparaginyl beta-hydroxylase	Q12797	7	7	14.2
Asporin	Q9BXN1	24	24	62.1
Astrocytic phosphoprotein PEA-15	Q15121	6	6	62.3
Atlastin-3	Q6DD88	16	16	48.8
ATP synthase subunit alpha, mitochondrial	P25705	14	14	37.1
ATP synthase subunit beta, mitochondrial	P06576	24	24	70.7
ATP synthase-coupling factor 6, mitochondrial	P18859	2	2	31.5
ATP-binding cassette sub-family F member 1	Q8NE71	2	2	2.6
ATP-citrate synthase	P53396	2	2	3.5
ATP-dependent RNA helicase DDX1	Q92499	2	2	4.7
Attractin	O75882	3	3	2.5
Autophagy-related protein 9B	Q674R7	2	2	4.3
Azurocidin	P20160	8	8	51.4
Bactericidal permeability-increasing protein	P17213	3	3	7
BAG family molecular chaperone regulator 3	O95817	2	2	5.6
Band 3 anion transport protein	P02730	35	35	64.5
Band 4.1-like protein 2	O43491	3	3	7.8
Barrier-to-autointegration factor	O75531	2	2	40.4
Basal cell adhesion molecule	P50895	17	17	43.8
Basement membrane-specific heparan sulfate proteoglycan core protein	P98160	172	172	59.8
Basigin	P35613	4	4	16.6
B-cell receptor-associated protein 31	P51572	7	7	31.3
Beta-2-glycoprotein 1	P02749	20	20	67
Beta-2-microglobulin	P61769	6	6	68.1
Beta-actin-like protein 2	Q562R1	15	3	41
Beta-hexosaminidase subunit alpha	P06865	2	2	7.2

Beta-hexosaminidase subunit beta	P07686	2	2	7.6
Beta-sarcoglycan	Q16585	2	2	13.8
Biglycan	P21810	29	28	60.9
Biliverdin reductase A	P53004	6	6	33.1
Bone marrow proteoglycan	P13727	2	2	12.6
Brain acid soluble protein 1	P80723	14	14	76.7
Branched-chain-amino-acid aminotransferase, cytosolic	P54687	4	4	20.2
BTB/POZ domain-containing protein KCTD12	Q96CX2	11	11	44.9
C4b-binding protein alpha chain	P04003	41	41	67.5
C4b-binding protein beta chain	P20851	6	6	26.6
Cadherin-13	P55290	17	17	27.8
Calcium-binding mitochondrial carrier protein SCaMC-1	Q6NUK1	3	3	10.7
Caldesmon	Q05682	67	66	60.9
Calmodulin	P62158	15	15	85.9
Calmodulin-like protein 5	Q9NZT1	3	3	30.8
Calnexin	P27824	22	22	45.9
Calpain small subunit 1	P04632	6	6	40.7
Calpain-1 catalytic subunit	P07384	4	4	11.6
Calpain-2 catalytic subunit	P17655	11	11	28.1
Calpastatin	P20810	10	10	26.4
Calponin-1	P51911	25	25	75.4
Calponin-2	Q99439	8	7	29.8
Calponin-3	Q15417	17	16	67.8
Calreticulin	P27797	25	25	79.4
Calumenin	O43852	17	17	62.9
cAMP-dependent protein kinase type I-alpha regulatory subunit	P10644	3	3	14.2
cAMP-dependent protein kinase type II-alpha regulatory subunit	P13861	7	7	27.5
Carbonic anhydrase 1	P00915	20	20	80.1
Carbonic anhydrase 2	P00918	19	19	71.2
Carbonyl reductase [NADPH] 1	P16152	5	3	30.7
Carboxypeptidase B2	Q96IY4	16	16	59.1
Carboxypeptidase N catalytic chain	P15169	3	3	7.4
Carboxypeptidase N subunit 2	P22792	9	9	32.1
Cartilage acidic protein 1	Q9NQ79	12	12	30.1
Cartilage oligomeric matrix protein	P49747	5	5	11.1
Caspase-14	P31944	6	6	35.5
Catalase	P04040	36	36	71
Catenin alpha-1	P35221	6	6	12.8
Cathelicidin antimicrobial peptide	P49913	5	5	28.8
Cathepsin B	P07858	17	17	50.4
Cathepsin D	P07339	29	29	66.5
Cathepsin G	P08311	8	8	30.6
Cathepsin H	P09668	3	3	12

Cathepsin L1	P07711	16	16	48.3
Cathepsin Z	Q9UBR2	11	11	43.9
Caveolin-1	Q03135	5	5	29.2
Caveolin-2	P51636	2	2	18.5
CD109 antigen	Q6YHK3	18	18	18.6
CD151 antigen	P48509	3	3	10.7
CD166 antigen	Q13740	10	10	29.3
CD177 antigen	Q8N6Q3	2	2	6.9
CD44 antigen	P16070	5	5	8
CD5 antigen-like	O43866	15	15	57.6
CD59 glycoprotein	P13987	6	6	28.1
CD63 antigen	P08962	2	2	8.4
CD9 antigen	P21926	4	4	25.4
CD97 antigen	P48960	2	2	4.3
Cell division control protein 42 homolog	P60953	7	6	38.2
Cell surface glycoprotein MUC18	P43121	16	16	35.9
Centromere protein F	P49454	2	2	0.7
Ceruloplasmin	P00450	60	60	71
cGMP-dependent protein kinase 1	Q13976	2	2	6
Charged multivesicular body protein 4b	Q9H444	2	2	10.7
Chloride intracellular channel protein 1	O00299	12	12	63.5
Chloride intracellular channel protein 4	Q9Y696	10	10	67.6
Choline transporter-like protein 2	Q8IWA5	3	3	5.9
Chondroitin sulfate proteoglycan 4	Q6UVK1	8	8	5.6
Chromobox protein homolog 3	Q13185	2	2	14.8
Chymase	P23946	5	5	31.6
Citrate synthase, mitochondrial	O75390	2	2	10.9
Clathrin heavy chain 1	Q00610	41	41	36.2
Clathrin light chain A	P09496	2	2	6.5
Clathrin light chain B	P09497	2	2	7.4
Clusterin	P10909	32	32	46.1
Coactosin-like protein	Q14019	7	7	54.9
Coagulation factor IX	P00740	14	14	42.1
Coagulation factor V	P12259	2	2	1.2
Coagulation factor VII	P08709	5	5	15.2
Coagulation factor X	P00742	11	11	25.6
Coagulation factor XII	P00748	8	8	21
Coagulation factor XIII A chain	P00488	28	27	53.1
Coagulation factor XIII B chain	P05160	4	4	7.1
Coatomer subunit beta	P48444	3	3	6.7
Coatomer subunit beta	P35606	2	2	5.5
Coatomer subunit delta	P53618	5	5	7.8
Coatomer subunit epsilon	O14579	3	3	19.5
Coatomer subunit gamma-1	Q9Y678	5	5	12.1

Cofilin-1	P23528	15	10	82.5
Cofilin-2	Q9Y281	7	2	44.6
Coiled-coil domain-containing protein 112	Q8NEF3	2	2	4
Coiled-coil domain-containing protein 18	Q5T9S5	2	2	1.8
Coiled-coil domain-containing protein 40	Q4G0X9	2	2	2.1
Coiled-coil domain-containing protein 80	Q76M96	2	2	3.1
Cold-inducible RNA-binding protein	Q14011	3	3	29.1
Collagen alpha-1(I) chain	P02452	20	20	14.8
Collagen alpha-1(III) chain	P02461	3	3	2.7
Collagen alpha-1(IV) chain	P02462	13	13	8.3
Collagen alpha-1(VI) chain	P12109	59	59	62.4
Collagen alpha-1(VIII) chain	P27658	6	6	13.6
Collagen alpha-1(XII) chain	Q99715	84	84	40.8
Collagen alpha-1(XIV) chain	Q05707	89	89	57.7
Collagen alpha-1(XV) chain	P39059	12	12	11.2
Collagen alpha-1(XVIII) chain	P39060	22	22	15.7
Collagen alpha-2(I) chain	P08123	14	14	9.6
Collagen alpha-2(IV) chain	P08572	17	17	15
Collagen alpha-2(V) chain	P05997	4	4	3.2
Collagen alpha-2(VI) chain	P12110	46	46	35.1
Collagen alpha-3(VI) chain	P12111	182	182	59.6
Collagen alpha-5(VI) chain	A8TX70	2	2	1.4
Collagen triple helix repeat-containing protein 1	Q96CG8	4	4	17.7
Complement C1q subcomponent subunit A	P02745	12	12	53.5
Complement C1q subcomponent subunit B	P02746	13	13	50.6
Complement C1q subcomponent subunit C	P02747	9	9	38.8
Complement C1q tumor necrosis factor-related protein 5	Q9BXJ0	5	5	34.6
Complement C1r subcomponent	P00736	23	22	45.1
Complement C1s subcomponen	P09871	25	25	49.9
Complement C2	P06681	9	9	18.2
Complement C3	P01024	155	142	84.5
Complement C4-A	P0C0L4	86	3	61.9
Complement C4-B	P0C0L5	88	5	63
Complement C5	P01031	80	80	60.7
Complement component 1 Q subcomponent-binding protein, mitochondrial	Q07021	2	2	5
Complement component C6	P13671	39	39	48.3
Complement component C7	P10643	30	30	52.3
Complement component C8 alpha chain	P07357	26	26	57.4
Complement component C8 beta chain	P07358	22	22	51.4
Complement component C8 gamma chain	P07360	9	9	59.9
Complement component C9	P02748	39	39	58.7
Complement decay-accelerating factor	P08174	3	3	15.2
Complement factor B	P00751	42	42	58.9
Complement factor D	P00746	7	7	41.1

Complement factor H	P08603	82	70	70.8
Complement factor H-related protein 1	Q03591	18	3	67.6
Complement factor H-related protein 2	P36980	8	2	43
Complement factor H-related protein 5	Q9BXR6	13	12	39.5
Complement factor I	P05156	9	9	19.6
Copine-1	Q99829	2	2	5.4
Copine-3	O75131	3	2	8.9
Core histone macro-H2A.1	O75367	5	5	19.1
Coronin-1A	P31146	13	13	42.1
Coronin-1B	Q9BR76	6	6	22.9
Coronin-1C	Q9ULV4	17	17	40.1
Corticosteroid-binding globulin	P08185	6	6	21
Costars family protein ABRACL	Q9P1F3	2	2	35.8
C-reactive protein	P02741	3	3	12.5
Creatine kinase B-type	P12277	8	8	38.1
C-type lectin domain family 11 member A	Q9Y240	6	6	22
C-type mannose receptor 2	Q9UBG0	3	3	5.7
Cullin-associated NEDD8-dissociated protein 1	Q86VP6	8	8	11.5
C-X-C motif chemokine 16	Q9H2A7	2	2	5.9
Cystatin-B	P04080	4	4	70.4
Cysteine and glycine-rich protein 1	P21291	12	12	67.4
Cysteine and glycine-rich protein 2	Q16527	11	11	66.8
Cysteine-rich protein 1	P50238	6	6	79.2
Cysteine-rich protein 2	P52943	8	8	69.2
Cytochrome b-245 heavy chain	P04839	2	2	4.7
Cytochrome b5 type B	O43169	3	3	32.2
Cytochrome c oxidase subunit 4 isoform 1, mitochondrial	P13073	2	2	10.7
Cytochrome c oxidase subunit 5A, mitochondrial	P20674	2	2	30
Cytoplasmic dynein 1 heavy chain 1	Q14204	11	11	4.6
Cytoplasmic dynein 1 intermediate chain 2	Q13409	4	3	9.9
Cytoplasmic dynein 1 light intermediate chain 2	O43237	4	3	12.2
Cytoplasmic FMR1-interacting protein 1	Q7L576	2	2	1.6
Cytoskeleton-associated protein 4	Q07065	14	14	35
Cytosol aminopeptidase	P28838	16	16	49.1
Cytosolic non-specific dipeptidase	Q96KP4	18	18	59.2
Decorin	P07585	25	24	52.9
Dehydrogenase/reductase SDR family member 7	Q9Y394	2	2	13.9
Delta(3,5)-Delta(2,4)-dienoyl-CoA isomerase, mitochondrial	Q13011	2	2	12.5
Delta-sarcoglycan	Q92629	2	2	7.3
Deoxynucleoside triphosphate triphosphohydrolase SAMHD1	Q9Y3Z3	15	15	36.4
Dermatopontin	Q07507	12	12	70.6
Dermcidin	P81605	2	2	20
Desmin	P17661	46	39	76.2

Destrin	P60981	17	16	83
Dihydrolipoyllysine-residue succinyltransferase component of 2-oxoglutarate dehydrogenase complex, mitochondrial	P36957	2	2	6.4
Dihydropyrimidinase-related protein 2	Q16555	28	24	68.2
Dihydropyrimidinase-related protein 3	Q14195	25	21	69.6
Dihydropyrimidine dehydrogenase [NADP(+)]	Q12882	2	2	2.2
Dipeptidyl peptidase 1	P53634	2	2	5.2
Dipeptidyl peptidase 2	Q9UHL4	2	2	6.9
DnaJ homolog subfamily C member 3	Q13217	2	2	7.9
Dolichyl-diphosphooligosaccharide--protein glycosyltransferase 48 kDa subunit	P39656	6	6	29.4
Dolichyl-diphosphooligosaccharide--protein glycosyltransferase subunit 1	P04843	16	16	40.2
Dolichyl-diphosphooligosaccharide--protein glycosyltransferase subunit 2	P04844	9	9	30.3
Dolichyl-diphosphooligosaccharide--protein glycosyltransferase subunit STT3A	P46977	2	2	5.2
Drebrin	Q16643	8	8	15.4
Drebrin-like protein	Q9UJU6	5	5	17.4
Dual specificity mitogen-activated protein kinase kinase 1	Q02750	2	2	12.2
Dynactin subunit 1	Q14203	5	5	8.9
Dynactin subunit 2	Q13561	5	5	18.2
Dynamin-2	P50570	2	2	2.9
Dynein heavy chain 2, axonemal	Q9P225	2	2	0.7
Dynein light chain 2, cytoplasmic	Q96FJ2	2	2	24.7
Dystroglycan	Q14118	2	2	4.9
Dystrophin	P11532	4	4	1.8
Early endosome antigen 1	Q15075	2	2	2.5
EF-hand domain-containing protein D1	Q9BUP0	6	5	37.2
EGF-containing fibulin-like extracellular matrix protein 1	Q12805	33	33	60.2
EGF-containing fibulin-like extracellular matrix protein 2	O95967	9	9	28
EGF-like repeat and discoidin I-like domain-containing protein 3	O43854	11	11	34.2
EH domain-containing protein 1	Q9H4M9	5	3	20.8
EH domain-containing protein 2	Q9NZN4	29	29	68.1
Elongation factor 1-alpha 1	P68104	24	24	68.8
Elongation factor 1-beta	P24534	5	5	37.8
Elongation factor 1-delta	P29692	6	6	29.9
Elongation factor 1-gamma	P26641	7	7	25.2
Elongation factor 2	P13639	32	32	47.2
Elongation factor Tu, mitochondrial	P49411	5	5	18.4
Emerin	P50402	2	2	11.4
EMILIN-1	Q9Y6C2	24	24	30.3

EMILIN-2	Q9BXX0	14	14	18.6
Endoglin	P17813	4	4	10
Endonuclease domain-containing 1 protein	O94919	2	2	9.2
Endoplasmic reticulum resident protein 29	P30040	6	6	33
Endoplasmic reticulum resident protein 44	Q9BS26	5	5	17
Endoplasmin	P14625	52	50	64.8
Eosinophil cationic protein	P12724	8	8	42.5
Epididymal secretory protein E1	P61916	3	3	31.8
Epsilon-sarcoglycan	O43556	3	3	14.2
Erlin-2	O94905	5	5	28.9
ERO1-like protein alpha	Q96HE7	6	6	16.7
Erythrocyte band 7 integral membrane protein	P27105	11	11	55.6
Erythrocyte membrane protein band 4.2	P16452	9	8	18.7
ES1 protein homolog, mitochondrial	P30042	3	3	22.4
Eukaryotic initiation factor 4A-I	P60842	15	8	44.8
Eukaryotic initiation factor 4A-II	Q14240	9	2	31.4
Eukaryotic translation initiation factor 2 subunit 1	P05198	2	2	9.2
Eukaryotic translation initiation factor 3 subunit H	O15372	2	2	8.5
Eukaryotic translation initiation factor 4B	P23588	4	4	11.6
Eukaryotic translation initiation factor 4H	Q15056	2	2	16.9
Eukaryotic translation initiation factor 5A-1	P63241	7	7	66.9
Exportin-1	O14980	3	3	4.6
Exportin-2	P55060	3	3	6.5
Extended synaptotagmin-1	Q9BSJ8	7	7	10.8
Extracellular matrix protein 1	Q16610	2	2	5.6
Extracellular sulfatase Sulf-1	Q8IWU6	6	6	12.9
Extracellular superoxide dismutase [Cu-Zn]	P08294	15	15	61.2
Ezrin	P15311	14	5	25.6
F-actin-capping protein subunit alpha-1	P52907	11	10	60.5
F-actin-capping protein subunit alpha-2	P47755	9	8	51
F-actin-capping protein subunit beta	P47756	11	11	52
Far upstream element-binding protein 2	Q92945	2	2	3.4
Fascin	Q16658	6	6	15.6
Fatty acid-binding protein, adipocyte	P15090	3	3	31.8
Fatty acid-binding protein, epidermal	Q01469	5	5	51.1
Fatty acid-binding protein, heart	P05413	2	2	12
Fermitin family homolog 2	Q96AC1	14	13	31.5
Fermitin family homolog 3	Q86UX7	14	14	28.6
Ferritin heavy chain	P02794	21	21	92.9
Ferritin light chain	P02792	13	13	60
Fibrillin-1	P35555	68	64	31.8
Fibrinogen alpha chain	P02671	58	58	48.2
Fibrinogen beta chain	P02675	59	59	82.1
Fibrinogen gamma chain	P02679	48	48	80.6

Fibroleukin	Q14314	7	7	26
Fibromodulin	Q06828	14	14	35.4
Fibronectin	P02751	143	143	68.4
Fibulin-1	P23142	31	31	45.2
Fibulin-2	P98095	26	26	25.9
Fibulin-5	Q9UBX5	18	18	39.5
Fibulin-7	Q53RD9	3	3	7.1
Ficolin-3	O75636	7	7	37.8
Filaggrin	P20930	4	4	6.5
Filamin-A	P21333	186	174	74.9
Filamin-B	O75369	23	13	12.8
Filamin-binding LIM protein 1	Q8WUP2	11	11	38.3
Filamin-C	Q14315	28	18	15.4
Flavin reductase (NADPH)	P30043	13	13	87.4
Folate receptor beta	P14207	2	2	14.1
Four and a half LIM domains protein 1	Q13642	23	23	56.7
Four and a half LIM domains protein 2	Q14192	9	9	39.4
Four and a half LIM domains protein 3	Q13643	5	5	28.2
Four and a half LIM domains protein 5	Q5TD97	3	3	10.9
Fructose-1,6-bisphosphatase 1	P09467	7	7	31.7
Fructose-bisphosphate aldolase A	P04075	31	27	86.5
Fructose-bisphosphate aldolase C	P09972	6	2	22.3
Fumarate hydratase, mitochondrial	P07954	3	3	16.7
Galectin-1	P09382	15	15	94.1
Galectin-3	P17931	4	4	20
Galectin-3-binding protein	Q08380	27	27	51.5
Gamma-enolase	P09104	5	2	22.1
Gamma-glutamylcyclotransferase	O75223	2	2	11.2
Gamma-glutamyltransferase 5	P36269	4	4	14.3
Gamma-interferon-inducible lysosomal thiol reductase	P13284	4	4	33.6
Gamma-parvin	Q9HBI0	2	2	8.5
Gamma-synuclein	O76070	5	5	54.3
Gap junction alpha-1 protein	P17302	2	2	8.1
Gelsolin	P06396	51	51	74.2
General vesicular transport factor p115	O60763	3	3	5
Glia-derived nexin	P07093	6	6	19.6
Glucose-6-phosphate 1-dehydrogenase	P11413	5	5	12.6
Glucose-6-phosphate isomerase	P06744	15	15	45.2
Glucosidase 2 subunit beta	P14314	12	12	25.4
Glucosylceramidase	P04062	2	2	9.7
Glutamate dehydrogenase 1, mitochondrial	P00367	15	15	39.6
Glutamine synthetase	P15104	5	5	16.6
Glutaredoxin-3	O76003	2	2	11.6
Glutathione peroxidase 1	P07203	3	3	26.6

Glutathione peroxidase 3	P22352	5	5	29.2
Glutathione reductase, mitochondrial	P00390	3	3	8.8
Glutathione S-transferase Mu 3	P21266	2	2	11.1
Glutathione S-transferase omega-1	P78417	8	8	36.9
Glutathione S-transferase P	P09211	11	11	69.5
Glyceraldehyde-3-phosphate dehydrogenase	P04406	33	33	91
Glycine--tRNA ligase	P41250	3	3	8.1
Glycogen phosphorylase, brain form	P11216	12	9	19.9
Glycogen phosphorylase, liver form	P06737	8	5	12.8
Glycogenin-1	P46976	4	4	15.1
Glycophorin-A	P02724	2	2	20.7
Glyoxalase domain-containing protein 4	Q9HC38	3	3	10.5
Glypican-6	Q9Y625	3	2	11.4
Golgi-associated plant pathogenesis-related protein 1	Q9H4G4	4	4	40.9
Granulins	P28799	5	5	8.9
Growth arrest-specific protein 6	Q14393	13	13	24.4
Growth factor receptor-bound protein 2	P62993	4	4	18.9
GTP-binding nuclear protein Ran	P62826	4	4	21.3
Guanine nucleotide-binding protein G(i) subunit alpha-2	P04899	14	10	57.2
Guanine nucleotide-binding protein G(I)/G(S)/G(T) subunit beta-1	P62873	12	6	55.9
Guanine nucleotide-binding protein G(I)/G(S)/G(T) subunit beta-2	P62879	14	7	61.8
Guanine nucleotide-binding protein G(s) subunit alpha isoforms short	P63092	9	8	28.4
Guanine nucleotide-binding protein subunit beta-2-like 1	P63244	4	4	22.7
Guanine nucleotide-binding protein subunit beta-4	Q9HAV0	8	2	31.5
Haptoglobin	P00738	32	15	67.7
Haptoglobin-related protein	P00739	27	10	79.3
Heat shock 70 kDa protein 1A/1B	P08107	38	29	63
Heat shock 70 kDa protein 4	P34932	6	6	11.5
Heat shock cognate 71 kDa protein	P11142	38	28	65
Heat shock protein beta-1	P04792	22	22	90.7
Heat shock protein beta-6	O14558	4	4	48.1
Heat shock protein beta-7	Q9UBY9	2	2	19.4
Heat shock protein HSP 90-alpha	P07900	36	25	59
Heat shock protein HSP 90-beta	P08238	34	21	57
Heat shock-related 70 kDa protein 2	P54652	13	4	21.6
Hematopoietic lineage cell-specific protein	P14317	4	4	11.1
Hemicentin-1	Q96RW7	19	19	5
Hemoglobin subunit alpha	P69905	14	10	91.5
Hemoglobin subunit beta	P68871	25	18	95.2
Hemoglobin subunit delta	P02042	15	8	95.2
Hemopexin	P02790	32	32	68
Heparin cofactor 2	P05546	19	19	45.5

Hepatoma-derived growth factor	P51858	7	7	45
Heterochromatin protein 1-binding protein 3	Q5SSJ5	3	3	8.5
Heterogeneous nuclear ribonucleoprotein A1	P09651	7	6	25.8
Heterogeneous nuclear ribonucleoprotein A3	P51991	4	4	16.7
Heterogeneous nuclear ribonucleoprotein D0	Q14103	7	6	25.1
Heterogeneous nuclear ribonucleoprotein F	P52597	5	3	22.7
Heterogeneous nuclear ribonucleoprotein H	P31943	5	3	17.6
Heterogeneous nuclear ribonucleoprotein H3	P31942	2	2	9
Heterogeneous nuclear ribonucleoprotein K	P61978	9	9	27
Heterogeneous nuclear ribonucleoprotein L	P14866	3	3	10.9
Heterogeneous nuclear ribonucleoprotein M	P52272	5	5	12.3
Heterogeneous nuclear ribonucleoprotein Q	O60506	2	2	5.5
Heterogeneous nuclear ribonucleoprotein U	Q00839	6	6	14.4
Heterogeneous nuclear ribonucleoproteins A2/B1	P22626	12	11	44.8
Heterogeneous nuclear ribonucleoproteins C1/C2	P07910	2	2	8.2
Hexokinase-1	P19367	5	4	6.5
Hexokinase-3	P52790	15	14	21.2
High mobility group nucleosome-binding domain-containing protein 4	O00479	4	4	48.9
High mobility group protein B1	P09429	9	8	40.5
Histidine-rich glycoprotein	P04196	18	18	33.7
Histone deacetylase 4	P56524	2	2	1.8
Histone deacetylase 9	Q9UKV0	2	2	2.1
Histone H1.0	P07305	3	3	18.6
Histone H1.2	P16403	10	3	31.9
Histone H1.4	P10412	10	3	35.2
Histone H1.5	P16401	4	3	17.7
Histone H2A type 1-C	Q93077	8	2	57.7
Histone H2A type 1-J	Q99878	8	2	58.6
Histone H2B type 1-M	Q99879	12	3	65.9
Histone H2B type 2-E	Q16778	11	2	65.9
Histone H3.3	P84243	5	2	41.2
Histone H4	P62805	7	7	51.5
HLA class II histocompatibility antigen gamma chain	P04233	5	5	22.3
HLA class II histocompatibility antigen, DP beta 1 chain	P04440	3	2	12.8
HLA class II histocompatibility antigen, DR alpha chain	P01903	9	8	33.1
HLA class II histocompatibility antigen, DR beta 4 chain	P13762	3	2	22.9
HLA class II histocompatibility antigen, DRB1-13 beta chain	Q5Y7A7	11	4	52.6
HLA class II histocompatibility antigen, DRB1-4 beta chain	P13760	10	3	57.1
Homeobox-containing protein 1	Q6NT76	2	2	6.4
Hornerin	Q86YZ3	6	6	7.7
Hsc70-interacting protein	P50502	8	8	20.6
Hsp90 co-chaperone Cdc37	Q16543	5	5	20.1
Hyaluronan and proteoglycan link protein 1	P10915	23	22	65.3

Hyaluronan and proteoglycan link protein 3	Q96S86	7	6	29.2
Hydroxyacyl-coenzyme A dehydrogenase, mitochondrial	Q16836	4	4	32.8
Hypoxia up-regulated protein 1	Q9Y4L1	8	8	14
Ig alpha-1 chain C region	P01876	22	8	59.8
Ig alpha-2 chain C region	P01877	18	4	66.8
Ig delta chain C region	P01880	3	3	15.6
Ig gamma-1 chain C region	P01857	28	16	70.9
Ig gamma-2 chain C region	P01859	20	9	70.6
Ig gamma-3 chain C region	P01860	20	9	66.6
Ig gamma-4 chain C region	P01861	18	11	70.9
Ig heavy chain V-I region HG3	P01743	3	3	43.6
Ig heavy chain V-I region V35	P23083	2	2	21.4
Ig heavy chain V-III region BRO	P01766	3	2	34.2
Ig heavy chain V-III region GAL	P01781	3	3	26.7
Ig heavy chain V-III region VH26	P01764	5	4	57.3
Ig kappa chain C region	P01834	14	14	97.2
Ig kappa chain V-I region EU	P01598	3	3	32.4
Ig kappa chain V-I region HK102	P01602	2	2	29.1
Ig kappa chain V-I region Ni	P01613	2	2	30.4
Ig kappa chain V-II region TEW	P01617	3	2	38.9
Ig kappa chain V-III region VG	P04433	2	2	26.1
Ig kappa chain V-IV region Len	P01700	3	2	23.2
Ig lambda chain V-III region LOI	P80748	2	2	21.6
Ig lambda chain V-III region SH	P01714	2	2	25
Ig lambda-2 chain C regions	P0CG05	14	3	93.4
Ig mu chain C region	P01871	35	13	63.1
Ig mu heavy chain disease protein	P04220	24	2	60.4
IgGFc-binding protein	Q9Y6R7	60	60	25.5
Immunoglobulin J chain	P01591	7	7	42.1
Immunoglobulin lambda-like polypeptide 5	B9A064	11	5	50
Immunoglobulin superfamily containing leucine-rich repeat protein	O14498	4	4	21
Importin subunit beta-1	Q14974	8	8	12.2
Importin-5	O00410	3	3	5.8
Inactive carboxypeptidase-like protein X2	Q8N436	7	6	16.5
Inactive phospholipase C-like protein 1	Q15111	2	2	3.3
Inorganic pyrophosphatase	Q15181	5	5	30.8
Insulin-like growth factor-binding protein 3	P17936	2	2	12
Insulin-like growth factor-binding protein 5	P24593	4	4	18.4
Insulin-like growth factor-binding protein 7	Q16270	14	14	58.5
Insulin-like growth factor-binding protein complex acid labile subunit	P35858	21	21	41
Integral membrane protein 2B	Q9Y287	4	4	23.7
Integrin alpha-1	P56199	20	20	24.3
Integrin alpha-3	P26006	5	5	5.6

Integrin alpha-5	P08648	5	5	8.4
Integrin alpha-7	Q13683	6	6	10.6
Integrin alpha-8	P53708	18	18	25.4
Integrin alpha-Iib	P08514	20	20	30
Integrin alpha-M	P11215	13	12	16.1
Integrin alpha-V	P06756	14	14	19
Integrin alpha-X	P20702	19	18	26.7
Integrin beta-1	P05556	31	31	45.1
Integrin beta-2	P05107	27	27	42.1
Integrin beta-3	P05106	13	13	28.6
Integrin beta-5	P18084	6	6	13.8
Integrin-linked protein kinase	Q13418	10	10	29.2
Intelectin-1	Q8WWA0	5	3	23.6
Inter-alpha-trypsin inhibitor heavy chain H1	P19827	30	30	45.9
Inter-alpha-trypsin inhibitor heavy chain H2	P19823	38	38	48.5
Inter-alpha-trypsin inhibitor heavy chain H3	Q06033	6	6	14.7
Inter-alpha-trypsin inhibitor heavy chain H4	Q14624	46	44	55.8
Inter-alpha-trypsin inhibitor heavy chain H5	Q86UX2	4	4	5.3
Interleukin-34	Q6ZMJ4	2	2	16.5
Interstitial collagenase	P03956	4	4	10.9
Isocitrate dehydrogenase [NAD] subunit alpha, mitochondrial	P50213	3	3	14.2
Isocitrate dehydrogenase [NADP] cytoplasmic	O75874	13	12	43.2
Isocitrate dehydrogenase [NADP], mitochondrial	P48735	5	4	14.6
Kallistatin	P29622	17	17	50.6
Katanin p60 ATPase-containing subunit A-like 2	Q8IYT4	2	2	3.7
Keratin, type I cytoskeletal 10	P13645	38	29	61.8
Keratin, type I cytoskeletal 14	P02533	15	4	34.5
Keratin, type I cytoskeletal 16	P08779	9	2	19.5
Keratin, type I cytoskeletal 17	Q04695	11	4	25.7
Keratin, type I cytoskeletal 18	P05783	13	7	39.3
Keratin, type I cytoskeletal 9	P35527	25	24	55.7
Keratin, type II cytoskeletal	P19013	4	2	8.2
Keratin, type II cytoskeletal 1	P04264	41	27	51.7
Keratin, type II cytoskeletal 2 epidermal	P35908	27	19	60.7
Keratin, type II cytoskeletal 5	P13647	18	4	26.3
Keratin, type II cytoskeletal 8	P05787	24	16	41.4
Kinectin	Q86UP2	6	6	7.1
Kinesin light chain 1	Q07866	2	2	4.5
Kinesin-1 heavy chain	P33176	5	5	7.4
Kininogen-1	P01042	26	25	36
KN motif and ankyrin repeat domain-containing protein 2	Q63ZY3	7	7	12.8
Lactadherin	Q08431	32	32	84
Lactotransferrin	P02788	52	49	73
Lactoylglutathione lyase	Q04760	5	5	34.2

Lamin-B1	P20700	14	13	30.7
Lamin-B2	Q03252	5	4	10.5
Laminin subunit alpha-2	P24043	3	3	1.6
Laminin subunit alpha-4	Q16363	4	4	3.9
Laminin subunit alpha-5	O15230	73	72	30.3
Laminin subunit beta-1	P07942	6	6	7.8
Laminin subunit beta-2	P55268	55	55	43.4
Laminin subunit gamma-1	P11047	49	49	38.3
L-amino-acid oxidase	Q96RQ9	9	9	19.2
Latent-transforming growth factor beta-binding protein 1	Q14766	20	19	17.1
Latent-transforming growth factor beta-binding protein 2	Q14767	28	27	22.5
Latent-transforming growth factor beta-binding protein 4	Q8N2S1	16	15	15.5
Legumain	Q99538	5	5	14.5
Leiomodin-1	P29536	15	15	31.8
Leucine-rich alpha-2-glycoprotein	P02750	10	10	42.4
Leucine-rich repeat flightless-interacting protein 1	Q32MZ4	7	7	14.6
Leucine-rich repeat-containing protein 17	Q8N6Y2	3	3	11.8
Leucine-rich repeat-containing protein 40	Q9H9A6	2	2	3.3
Leucine-rich repeat-containing protein 59	Q96AG4	2	2	8.1
Leukocyte elastase inhibitor	P30740	5	4	17.9
Leukotriene A-4 hydrolase	P09960	12	12	30.1
LIM and calponin homology domains-containing protein 1	Q9UPQ0	3	3	4.5
LIM and cysteine-rich domains protein 1	Q9NZU5	19	19	58.4
LIM and senescent cell antigen-like-containing domain protein 1	P48059	7	5	25.5
LIM and senescent cell antigen-like-containing domain protein 2	Q7Z4I7	5	3	18.5
LIM and SH3 domain protein 1	Q14847	15	15	47.1
LIM domain-binding protein 3	O75112	3	3	8.3
Lipoma-preferred partner	Q93052	23	23	63.2
Lipopolysaccharide-binding protein	P18428	15	15	33.3
Liver carboxylesterase 1	P23141	12	12	24.5
L-lactate dehydrogenase A chain	P00338	21	20	78.6
L-lactate dehydrogenase B chain	P07195	13	12	44.9
Lumican	P51884	28	28	57.4
Lupus La protein	P05455	5	5	16.7
Lymphocyte-specific protein 1	P33241	12	12	59.6
Lysosomal acid lipase/cholesteryl ester hydrolase	P38571	9	9	35.6
Lysosomal alpha-glucosidase	P10253	8	8	12.8
Lysosomal protective protein	P10619	2	2	5
Lysosome membrane protein 2	Q14108	3	3	9.8
Lysosome-associated membrane glycoprotein 1	P11279	4	4	10.6
Lysozyme C	P61626	15	15	68.2
Lysyl oxidase homolog 1	Q08397	5	5	17.4

Macrophage mannose receptor 1	P22897	2	2	4
Macrophage metalloelastase	P39900	23	23	42.8
Macrophage migration inhibitory factor	P14174	2	2	17.4
Macrophage receptor MARCO	Q9UEW3	4	4	8.7
Macrophage scavenger receptor types I and II	P21757	2	2	8.4
Macrophage-capping protein	P40121	16	16	56.6
Macrosialin	P34810	2	2	15.3
Major vault protein	Q14764	21	21	37.5
Malate dehydrogenase, cytoplasmic	P40925	4	4	17.7
Malate dehydrogenase, mitochondrial	P40926	3	3	12.1
MAM domain-containing protein 2	Q7Z304	4	4	6.4
Mannosyl-oligosaccharide 1,2-alpha-mannosidase IA	P33908	7	7	17.8
Marginal zone B- and B1-cell-specific protein	Q8WU39	2	2	21.2
Mast cell carboxypeptidase A	P15088	2	2	4.3
Matrilysin	P09237	4	4	24.7
Matrin-3	P43243	2	2	5.4
Matrix Gla protein	P08493	5	5	39.8
Matrix metalloproteinase-9	P14780	15	15	31.1
Matrix-remodeling-associated protein 5	Q9NR99	7	7	3.4
Matrix-remodeling-associated protein 7	P84157	2	2	19.1
Membrane primary amine oxidase	Q16853	27	27	48.2
Membrane-associated progesterone receptor component 1	O00264	4	4	34.9
Metalloproteinase inhibitor 1	P01033	8	8	54.6
Metalloproteinase inhibitor 3	P35625	10	10	53.1
Metallothionein-2	P02795	4	2	67.2
Microfibril-associated glycoprotein 4	P55083	13	13	57.6
Microfibrillar-associated protein 5	Q13361	5	5	27.2
Microsomal glutathione S-transferase 3	O14880	3	3	40.8
Microtubule-associated protein 1B	P46821	37	37	26.2
Microtubule-associated protein 4	P27816	12	12	18.9
Mimecan	P20774	27	27	57
Mitochondrial inner membrane protein	Q16891	5	5	12.1
Mitogen-activated protein kinase 1	P28482	3	3	16.7
Moesin	P26038	45	36	71.1
Monocyte differentiation antigen CD14	P08571	13	13	45.6
Multimerin-2	Q9H8L6	2	2	5.4
Musculoskeletal embryonic nuclear protein 1	Q8IVN3	3	3	46.3
Myeloblastin	P24158	4	4	27.7
Myeloid cell nuclear differentiation antigen	P41218	3	3	7.4
Myeloperoxidase	P05164	32	30	47.4
Myoferlin	Q9NZM1	8	8	6.2
Myosin light chain 3	P08590	7	7	41.5
Myosin light chain kinase, smooth muscle	Q15746	17	17	13.4
Myosin light polypeptide 6	P60660	17	17	88.1

Myosin phosphatase Rho-interacting protein	Q6WCQ1	3	3	4.8
Myosin regulatory light chain 12B	O14950	13	6	80.2
Myosin regulatory light chain 2, ventricular/cardiac muscle isoform	P10916	5	5	37.3
Myosin regulatory light polypeptide 9	P24844	15	8	66.3
Myosin-10	P35580	173	142	68.7
Myosin-11	P35749	169	144	67.1
Myosin-7	P12883	5	4	3.6
Myosin-9	P35579	151	122	64.4
Myotrophin	P58546	5	5	64.4
Myozenin-1	Q9NP98	4	4	27.4
Myristoylated alanine-rich C-kinase substrate	P29966	11	11	49.4
N-acetyl-D-glucosamine kinase	Q9UJ70	5	5	20.6
N-acetylmuramoyl-L-alanine amidase	Q96PD5	20	20	62.7
NADH-cytochrome b5 reductase 3	P00387	16	16	74.4
Nascent polypeptide-associated complex subunit alpha	Q13765	6	6	34
Nebulin	P20929	4	4	0.6
Nephronectin	Q6UXI9	5	5	9.6
Neuroblast differentiation-associated protein AHNAK	Q09666	121	121	44.4
Neuromodulin	P17677	2	2	16.4
Neutral alpha-glucosidase AB	Q14697	16	16	30.7
Neutral cholesterol ester hydrolase 1	Q6PIU2	3	3	14.2
Neutrophil cytosol factor 2	P19878	2	2	6.1
Neutrophil defensin 3	P59666	4	4	20.2
Neutrophil elastase	P08246	3	3	16.9
Neutrophil gelatinase-associated lipocalin	P80188	5	5	39.4
Nexilin	Q0ZGT2	19	19	28.3
Niban-like protein 1	Q96TA1	8	8	18.1
Nicotinamide N-methyltransferase	P40261	2	2	15.5
Nicotinamide phosphoribosyltransferase	P43490	7	7	25.5
Nidogen-1	P14543	29	28	35
Nidogen-2	Q14112	13	12	14.4
Non-histone chromosomal protein HMG-17	P05204	5	5	64.4
Nuclear mitotic apparatus protein 1	Q14980	2	2	1.1
Nuclease-sensitive element-binding protein 1	P67809	8	7	42.3
Nucleobindin-1	Q02818	5	5	14.5
Nucleolin	P19338	14	14	24.5
Nucleophosmin	P06748	9	9	42.2
Nucleoside diphosphate kinase B	P22392	5	3	43.4
Nucleosome assembly protein 1-like 1	P55209	3	3	11.8
Nucleosome assembly protein 1-like 4	Q99733	5	5	19.5
Obg-like ATPase 1	Q9NTK5	2	2	9.3
Olfactomedin-like protein 1	Q6UWY5	2	2	8.7
Osteomodulin	Q99983	4	4	15.7
Osteopontin	P10451	12	12	58

Palladin	Q8WX93	24	24	25.5
Palmitoyl-protein thioesterase 1	P50897	8	8	44.8
Parathymosin	P20962	2	2	12.7
PDZ and LIM domain protein 1	O00151	11	11	62.3
PDZ and LIM domain protein 3	Q53GG5	10	10	38.2
PDZ and LIM domain protein 4	P50479	3	3	19.4
PDZ and LIM domain protein 5	Q96HC4	11	11	28.7
PDZ and LIM domain protein 7	Q9NR12	18	18	53.4
Peptidoglycan recognition protein 1	O75594	2	2	19.9
Peptidyl-prolyl cis-trans isomerase A	P62937	12	12	74.5
Peptidyl-prolyl cis-trans isomerase B	P23284	14	14	57.4
Peptidyl-prolyl cis-trans isomerase FKBP1A	P62942	4	4	41.7
Perilipin-2	Q99541	18	18	56.3
Perilipin-3	O60664	8	8	35.3
Periostin	Q15063	56	56	59.4
Peroxiredoxin-1	Q06830	21	17	76.9
Peroxiredoxin-2	P32119	23	22	90.4
Peroxiredoxin-4	Q13162	10	7	49.4
Peroxiredoxin-6	P30041	12	12	70.5
Peroxisomal multifunctional enzyme type 2	P51659	6	6	15.2
Phosphatidate cytidylyltransferase 2	O95674	2	2	6.5
Phosphatidylethanolamine-binding protein 1	P30086	11	11	74.9
Phosphatidylinositol-binding clathrin assembly protein	Q13492	3	3	9
Phosphatidylinositol-glycan-specific phospholipase D	P80108	12	12	20.5
Phosphoglucomutase-1	P36871	13	13	43.1
Phosphoglucomutase-2	Q96G03	3	3	10.1
Phosphoglucomutase-like protein 5	Q15124	27	27	63
Phosphoglycerate kinase 1	P00558	35	29	85.6
Phosphoglycerate mutase 1	P18669	12	7	59.8
Phospholipase A2, membrane associated	P14555	5	5	30.6
Phospholipase D3	Q8IV08	13	13	34.7
Phospholipid transfer protein	P55058	25	25	56.4
Pigment epithelium-derived factor	P36955	23	23	64.6
Plasma kallikrein	P03952	27	27	52.8
Plasma membrane calcium-transporting ATPase 4	P23634	3	2	4
Plasma protease C1 inhibitor	P05155	20	20	39
Plasma serine protease inhibitor	P05154	3	3	8.6
Plasminogen	P05121	6	6	21.4
Plasminogen activator inhibitor 1	P13796	40	34	79.1
Plasminogen activator inhibitor 1 RNA-binding protein	Q8NC51	3	3	11
Plastin-2	P00747	63	63	78.1
Plastin-3	P13797	44	37	72.9
Platelet glycoprotein 4	P16671	10	10	29.9
Platelet glycoprotein Ib beta chain	P13224	2	2	10.7

Platelet-activating factor acetylhydrolase	Q13093	3	3	12
Platelet-activating factor acetylhydrolase IB subunit alpha	P43034	2	2	6.3
Platelet-activating factor acetylhydrolase IB subunit beta	P68402	5	5	48.9
Pleckstrin	P08567	4	4	20.6
Pleckstrin homology domain-containing family O member 2	Q8TD55	2	2	7.3
Plectin	Q15149	151	151	39.8
Plexin domain-containing protein 2	Q6UX71	5	5	13.4
Poly(rC)-binding protein 1	Q15365	5	3	25.6
Polycystic kidney disease protein 1-like 1	Q8TDX9	2	2	0.7
Polymerase I and transcript release factor	Q6NZI2	20	20	40.3
Polypyrimidine tract-binding protein 1	P26599	2	2	11.3
Pregnancy zone protein	P20742	31	23	28
Prelamin-A/C	P02545	70	69	83.4
Prenylcysteine oxidase 1	Q9UHG3	17	17	46.9
PRKC apoptosis WT1 regulator protein	Q96IZ0	5	5	25.6
Proactivator polypeptide	P07602	14	14	35.5
Procollagen C-endopeptidase enhancer 1	Q15113	4	4	14.6
Procollagen C-endopeptidase enhancer 2	Q9UKZ9	7	7	20.2
Procollagen-lysine,2-oxoglutarate 5-dioxygenase 1	Q02809	15	15	29.8
Profilin-1	P07737	10	10	63.6
Profilin-2	P35080	3	3	26.4
Programmed cell death 6-interacting protein	Q8WUM4	9	9	16.9
Prolargin	P51888	28	28	53.7
Prolow-density lipoprotein receptor-related protein 1	Q07954	29	29	9.6
Prolyl 4-hydroxylase subunit alpha-1	P13674	3	3	8.4
Properdin	P27918	3	3	8.5
Prostacyclin synthase	Q16647	9	9	25.4
Prostaglandin E synthase 3	Q15185	3	3	26.2
Prostaglandin reductase 1	Q14914	2	2	9.1
Prostaglandin-H2 D-isomerase	P41222	2	2	17.4
Proteasome activator complex subunit 1	Q06323	10	10	45.4
Proteasome activator complex subunit 2	Q9UL46	7	7	43.5
Proteasome subunit alpha type-3	P25788	4	4	17.6
Proteasome subunit alpha type-4	P25789	3	3	22.2
Proteasome subunit beta type-1	P20618	2	2	17
Protein 4.1	P11171	5	5	11.1
Protein AMBP	P02760	23	23	58.8
Protein broad-minded	Q96NH3	2	2	2.7
Protein CDV3 homolog	Q9UKY7	2	2	31.4
Protein disulfide-isomerase	P07237	43	43	76.4
Protein disulfide-isomerase A3	P30101	28	28	58.8
Protein disulfide-isomerase A4	P13667	13	13	27
Protein disulfide-isomerase A6	Q15084	12	12	40.5

Protein DJ-1	Q99497	5	5	48.7
Protein enabled homolog	Q8N8S7	2	2	8.5
Protein ERGIC-53	P49257	3	3	13.3
Protein FAM49B	Q9NUQ9	4	4	15.7
Protein farnesyltransferase/geranylgeranyltransferase type-1 subunit alpha	P49354	2	2	8.2
Protein KIAA1199	Q8WUJ3	4	4	4
Protein kinase C delta-binding protein	Q969G5	8	8	34.5
Protein MRVI1	Q9Y6F6	2	2	3.7
Protein names	Q92597	9	9	45.2
Protein Niban	Q9BZQ8	14	14	25
Protein NOV homolog	P48745	4	4	14.8
Protein NOXP20	Q8IWE2	4	4	12.1
Protein phosphatase 1 regulatory subunit 12A	O14974	5	5	6.5
Protein phosphatase 1 regulatory subunit 12B	O60237	2	2	3
Protein phosphatase 1 regulatory subunit 14A	Q96A00	2	2	18.4
Protein PML	P29590	2	2	2.4
Protein PTHB1	Q3SYG4	2	2	4.7
Protein S100-A10	P60903	6	6	63.9
Protein S100-A11	P31949	10	10	86.7
Protein S100-A12	P80511	7	7	51.1
Protein S100-A4	P26447	10	10	64.4
Protein S100-A6	P06703	6	6	90
Protein S100-A7	P31151	3	3	34.7
Protein S100-A8	P05109	18	18	100
Protein S100-A9	P06702	11	11	85.1
Protein S100-P	P25815	2	2	17.9
Protein SEC13 homolog	P55735	2	2	10.6
Protein SET	Q01105	5	5	27.6
Protein transport protein Sec23A	Q15436	2	2	5.2
Protein Z-dependent protease inhibitor	Q9UK55	9	9	27.9
Protein-glutamine gamma-glutamyltransferase 2	P21980	38	38	63.6
Protein-L-isoaspartate(D-aspartate) O-methyltransferase	P22061	2	2	18.9
Proteoglycan 4	Q92954	4	4	4.8
Prothrombin	P00734	41	41	58.7
Prothymosin alpha	P06454	4	4	34.2
Protocadherin-23	Q6V1P9	2	2	1.3
Purine nucleoside phosphorylase	P00491	4	4	26.3
Putative ankyrin repeat domain-containing protein 31	Q8N7Z5	3	3	2.1
Putative eukaryotic translation initiation factor 2 subunit 3-like protein	Q2VIR3	2	2	11.2
Putative heat shock protein HSP 90-beta 2	Q58FF8	5	2	13.6
Putative RNA-binding protein 3	P98179	4	4	35
Pyridoxal kinase	O00764	7	7	25.3
Pyruvate kinase PKM	P14618	50	50	86.6

Rab GDP dissociation inhibitor alpha	P31150	15	9	48.3
Rab GDP dissociation inhibitor beta	P50395	18	12	53
Ras GTPase-activating-like protein IQGAP1	P46940	48	47	44.7
Ras GTPase-activating-like protein IQGAP2	Q13576	3	2	3.7
Ras suppressor protein 1	Q15404	15	15	66.4
Ras-related C3 botulinum toxin substrate 1	P63000	6	2	33.9
Ras-related protein Rab-10	P61026	3	2	17
Ras-related protein Rab-14	P61106	2	2	20.9
Ras-related protein Rab-1A	P62820	5	4	32.2
Ras-related protein Rab-2A	P61019	3	2	14.6
Ras-related protein Rab-5C	P51148	2	2	12
Ras-related protein Rab-6B	Q9NRW1	2	2	12
Ras-related protein Rab-7a	P51149	2	2	11.6
Ras-related protein Ral-A	P11233	3	3	20.4
Ras-related protein Rap-1b	P61224	8	2	57.1
Ras-related protein R-Ras	P10301	5	5	32.6
Receptor expression-enhancing protein 5	Q00765	2	2	11.1
Receptor-type tyrosine-protein phosphatase C	P08575	2	2	2
Reticulocalbin-1	Q15293	9	9	49.5
Reticulocalbin-3	Q96D15	5	5	17.1
Reticulon-4	Q9NQC3	8	8	12.1
Retinal dehydrogenase 1	P00352	8	7	23
Retinoid-inducible serine carboxypeptidase	Q9HB40	3	3	9.3
Retinol-binding protein 4	P02753	9	9	72.6
Rho GDP-dissociation inhibitor 1	P52565	11	11	79.4
Rho GDP-dissociation inhibitor 2	P52566	13	13	73.1
Rho GTPase-activating protein 1	Q07960	12	12	42.4
Rho GTPase-activating protein 18	Q8N392	2	2	5.1
Rho GTPase-activating protein 8	P85298	2	2	4.1
Rho-associated protein kinase 2	O75116	2	2	1.9
Ribonuclease 4	P34096	8	8	49.7
Ribonuclease inhibitor	P13489	12	12	42.3
Ribonuclease pancreatic	P07998	5	5	59
Ribosome-binding protein 1	Q9P2E9	15	15	14.5
Ryanodine receptor 3	Q15413	2	2	0.4
Sarcolemmal membrane-associated protein	Q14BN4	4	4	5.8
Sarcoplasmic/endoplasmic reticulum calcium ATPase 2	P16615	4	4	7.4
Scavenger receptor cysteine-rich type 1 protein M130	Q86VB7	15	15	18.8
Scavenger receptor cysteine-rich type 1 protein M160	Q9NR16	2	2	2.9
Secernin-1	Q12765	6	6	20.3
Secreted frizzled-related protein 2	Q96HF1	2	2	8.8
Secreted frizzled-related protein 3	Q92765	6	6	19.4
Secreted frizzled-related protein 4	Q6FHJ7	3	3	13
Secreted phosphoprotein 24	Q13103	7	7	28.9

Selenium-binding protein 1	Q13228	10	10	35.2
Selenoprotein P	P49908	3	3	10.5
Semaphorin-3C	Q99985	3	3	4.7
Seprase	Q12884	2	2	3.7
Septin-11	Q9NVA2	6	5	17.9
Septin-2	Q15019	12	12	54.8
Septin-7	Q16181	12	11	36.6
Septin-8	Q92599	4	3	12.2
Septin-9	Q9UHD8	2	2	8.2
Serine protease 23	O95084	3	3	8.4
Serine protease HTRA1	Q92743	29	29	61
Serine/arginine-rich splicing factor 1	Q07955	3	3	10.5
Serine/arginine-rich splicing factor 2	Q01130	4	4	21.7
Serine/arginine-rich splicing factor 3	P84103	3	2	18.3
Serine/threonine-protein phosphatase 2A 65 kDa regulatory subunit A alpha isoform	P30153	10	10	25.3
Serotransferrin	P02787	83	80	79.4
Serpin B6	P35237	4	4	18.6
Serpin H1	P50454	16	16	46.9
Serum albumin	P02768	116	109	94.4
Serum amyloid A-4 protein	P35542	7	7	43.8
Serum amyloid P-component	P02743	16	16	50.7
Serum paraoxonase/arylesterase 1	P27169	23	23	72.4
S-formylglutathione hydrolase	P10768	10	10	67.7
SH3 domain-binding glutamic acid-rich-like protein	O75368	10	10	85.1
SH3 domain-binding glutamic acid-rich-like protein 3	Q9H299	4	4	39.8
Sialic acid synthase	Q9NR45	2	2	10.6
Signal transducer and activator of transcription 1-alpha/beta	P42224	3	3	5.9
Signal transducer and activator of transcription 3	P40763	3	3	7.9
Smoothelin	P53814	9	9	13.6
Sodium/potassium-transporting ATPase subunit alpha-1	P05023	11	11	16.7
Sodium/potassium-transporting ATPase subunit beta-3	P54709	4	4	24.4
Solute carrier family 2, facilitated glucose transporter member 1	P11166	7	7	22
Solute carrier family 2, facilitated glucose transporter member 3	P11169	3	3	8.1
Solute carrier family 22 member 20	A6NK97	2	2	4.9
Somatomedin-B and thrombospondin type-1 domain-containing protein	Q8IVN8	7	7	45.5
Sorbin and SH3 domain-containing protein 1	Q9BX66	24	23	22.2
Sorbin and SH3 domain-containing protein 2	O94875	19	18	24.4
Sorting nexin-2	O60749	3	2	6.6
Sorting nexin-6	Q9UNH7	2	2	4.7
SPARC-like protein 1	Q14515	9	9	24.5

SPARC-related modular calcium-binding protein 2	Q9H3U7	3	3	12.8
Spectrin alpha chain, erythrocytic 1	P02549	71	71	40.6
Spectrin alpha chain, non-erythrocytic 1	Q13813	57	57	33.7
Spectrin beta chain, erythrocytic	P11277	64	60	41.1
Spectrin beta chain, non-erythrocytic 1	Q01082	30	26	20.3
Spectrin beta chain, non-erythrocytic 4	Q9H254	4	2	1.4
S-phase kinase-associated protein 1	P63208	3	3	25.2
Spliceosome RNA helicase DDX39B	Q13838	2	2	7
Src substrate cortactin	Q14247	10	10	26.9
Stabilin-1	Q9NY15	13	13	7.2
Staphylococcal nuclease domain-containing protein 1	Q7KZF4	5	5	13
Stathmin	P16949	2	2	15.4
Stress-70 protein, mitochondrial	P38646	12	12	23.6
Stress-induced-phosphoprotein 1	P31948	10	10	21.7
Succinate dehydrogenase [ubiquinone] flavoprotein subunit, mitochondrial	P31040	3	3	9.8
Sulfhydryl oxidase 1	O00391	12	12	20.3
Sulfide:quinone oxidoreductase, mitochondrial	Q9Y6N5	3	3	8.4
SUN domain-containing protein 2	Q9UH99	6	6	13.7
Superkiller viralicidic activity 2-like 2	P42285	3	3	3.7
Superoxide dismutase [Cu-Zn]	P00441	12	12	87
Superoxide dismutase [Mn], mitochondrial	P04179	11	11	76.6
Sushi domain-containing protein 2	Q9UGT4	11	11	32.8
Synaptic vesicle membrane protein VAT-1 homolog	Q99536	11	11	50.6
Synaptopodin	Q8N3V7	4	4	7.5
Synaptopodin-2	Q9UMS6	15	15	22
Synemin	O15061	8	8	7
Syntaxin-7	O15400	3	3	14.9
Talin-1	Q9Y490	132	120	71
Talin-2	Q9Y4G6	15	3	6.9
Target of Myb protein 1	O60784	2	2	5.9
Target of Nesh-SH3	Q7Z7G0	18	18	24.6
T-complex protein 1 subunit alpha	P17987	6	6	20.1
T-complex protein 1 subunit beta	P78371	5	5	14.2
T-complex protein 1 subunit delta	P50991	4	4	13.5
T-complex protein 1 subunit epsilon	P48643	4	4	13.3
T-complex protein 1 subunit eta	Q99832	3	3	7.6
T-complex protein 1 subunit gamma	P49368	5	5	13.4
T-complex protein 1 subunit theta	P50990	7	7	16.8
T-complex protein 1 subunit zeta	P40227	5	5	16.2
Tenascin	P24821	113	113	56.3
Tenascin-X	P22105	86	86	33.6
Tensin-1	Q9HBL0	36	34	40.1
Tensin-3	Q68CZ2	4	2	4.4
Testin	Q9UGI8	10	10	30.6

Tetranectin	P05452	11	8	65.8
Thioredoxin	P10599	4	4	45.7
Thioredoxin domain-containing protein 5	Q8NBS9	10	10	32.9
Thioredoxin reductase 1, cytoplasmic	Q16881	3	3	9.7
Thioredoxin-dependent peroxide reductase, mitochondrial	P30048	5	5	29.7
Thioredoxin-like protein 1	O43396	2	2	11.4
Thioredoxin-related transmembrane protein 4	Q9H1E5	2	2	8.6
Thrombospondin type-1 domain-containing protein 4	Q6ZMP0	3	3	3
Thrombospondin-1	P07996	68	22	57.4
Thrombospondin-2	P35442	52	49	48.3
Thy-1 membrane glycoprotein	P04216	3	3	16.1
Thymidine phosphorylase	P19971	12	12	38.6
Thymosin beta-10	P63313	4	3	68.2
Thymosin beta-4	P62328	10	9	93.2
Thyroid receptor-interacting protein 6	Q15654	3	3	16.6
Thyroxine-binding globulin	P05543	6	6	26
Titin	Q8WZ42	7	7	0.3
Transaldolase	P37837	11	11	33.8
Transcriptional activator protein Pur-alpha	Q00577	4	4	25.8
Transferrin receptor protein 1	P02786	3	3	5.5
Transforming growth factor beta-1-induced transcript 1 protein	O43294	25	25	87.6
Transforming growth factor-beta-induced protein ig-h3	Q15582	43	43	71.4
Transforming protein RhoA	P61586	9	9	56
Transgelin	Q01995	29	28	91
Transgelin-2	P37802	24	23	95
Transitional endoplasmic reticulum ATPase	P55072	32	32	51.5
Transketolase	P29401	16	16	41.7
Translationally-controlled tumor protein	P13693	7	7	50
Transmembrane emp24 domain-containing protein 10	P49755	4	4	18.3
Transmembrane emp24 domain-containing protein 2	Q15363	5	5	30.3
Transmembrane emp24 domain-containing protein 9	Q9BVK6	4	4	20.4
Transmembrane glycoprotein NMB	Q14956	6	6	8.2
Transmembrane protein 43	Q9BTV4	3	3	17
Transthyretin	P02766	11	11	69.4
Trifunctional enzyme subunit alpha, mitochondrial	P40939	15	15	32.4
Trifunctional enzyme subunit beta, mitochondrial	P55084	2	2	6.1
Triosephosphate isomerase	P60174	16	16	69.2
Tripeptidyl-peptidase 1	O14773	5	5	15.5
tRNA-splicing ligase RtcB homolog	Q9Y3I0	3	3	13.1
Tropomodulin-1	P28289	2	2	10.3
Tropomodulin-3	Q9NYL9	3	3	12.5
Tropomyosin alpha-1 chain	P09493	54	27	89.4
Tropomyosin alpha-3 chain	P06753	30	7	60

Tropomyosin alpha-4 chain	P67936	43	24	87.1
Tropomyosin beta chain	P07951	52	5	79.2
Tryptase alpha/beta-1	Q15661	5	5	23.6
Tryptophan--tRNA ligase, cytoplasmic	P23381	9	9	28.9
Tubulin alpha-4A chain	P68366	24	4	61.2
Tubulin beta chain	P07437	31	6	73.4
Tubulin beta-1 chain	Q9H4B7	6	2	14.2
Tubulin beta-2B chain	Q9BVA1	29	4	75.1
Tubulin beta-6 chain	Q9BUF5	19	7	62.8
Tubulin beta-8 chain	Q3ZCM7	10	2	22.7
Tubulin polymerization-promoting protein family member 3	Q9BW30	2	2	16.5
Tubulointerstitial nephritis antigen-like	Q9GZM7	29	29	77.3
Tumor necrosis factor receptor superfamily member 11B	O00300	7	7	23.9
Tumor protein D54	O43399	7	7	40.8
Twinfilin-2	Q6IBS0	5	5	23.2
Tyrosine-protein phosphatase non-receptor type 6	P29350	2	2	6.2
Ubiquitin carboxyl-terminal hydrolase 14	P54578	3	3	7.1
Ubiquitin carboxyl-terminal hydrolase 5	P45974	8	8	19.7
Ubiquitin carboxyl-terminal hydrolase isozyme L1	P09936	7	7	43.9
Ubiquitin-60S ribosomal protein L40	P62987	5	5	38.3
Ubiquitin-associated domain-containing protein 1	Q9BSL1	2	2	9.6
Ubiquitin-conjugating enzyme E2 L3	P68036	5	5	50
Ubiquitin-conjugating enzyme E2 N	P61088	2	2	17.1
Ubiquitin-like modifier-activating enzyme 1	P22314	25	25	42.6
UDP-glucose:glycoprotein glucosyltransferase 1	Q9NYU2	6	6	7.8
UMP-CMP kinase	P30085	7	7	43.9
Unconventional myosin-Ic	O00159	3	3	3.6
Unconventional myosin-Id	O94832	6	6	8.3
Unconventional myosin-Ie	Q12965	3	3	3.6
UTP--glucose-1-phosphate uridylyltransferase	Q16851	4	4	16.7
Utrophin	P46939	7	7	4.3
UV excision repair protein RAD23 homolog A	P54725	2	2	16.5
UV excision repair protein RAD23 homolog B	P54727	4	4	16.9
Vacuolar protein sorting-associated protein 35	Q96QK1	11	11	16.8
Vasodilator-stimulated phosphoprotein	P50552	4	4	10.8
Versican core protein	P13611	48	48	13.2
Very long-chain specific acyl-CoA dehydrogenase, mitochondrial	P49748	2	2	5.5
Vesicle-associated membrane protein-associated protein A	Q9P0L0	3	3	18.1
Vesicular integral-membrane protein VIP36	Q12907	3	3	14.3
Vimentin	P08670	87	79	97.4
Vinculin	P18206	92	92	75.4
Vitamin D-binding protein	P02774	36	36	75.5

Vitamin K-dependent protein C	P04070	9	9	30.2
Vitamin K-dependent protein S	P07225	21	21	36.1
Vitamin K-dependent protein Z	P22891	3	3	12.5
Vitronectin	P04004	26	26	46.2
Voltage-dependent anion-selective channel protein 1	P21796	3	3	15.2
Voltage-dependent anion-selective channel protein 2	P45880	10	10	54.4
Voltage-dependent calcium channel subunit alpha-2/delta-1	P54289	9	9	13.4
von Willebrand factor	P04275	16	16	8.1
V-type proton ATPase 116 kDa subunit a isoform 3	Q13488	2	2	6.6
V-type proton ATPase catalytic subunit A	P38606	13	13	31.6
V-type proton ATPase subunit B, brain isoform	P21281	8	8	24.1
WD repeat and FYVE domain-containing protein 3	Q8IZQ1	2	2	1.2
WD repeat-containing protein 1	O75083	28	28	67
WNT1-inducible-signaling pathway protein 2	O76076	3	3	17.6
Xaa-Pro dipeptidase	P12955	5	5	13.2
Xin actin-binding repeat-containing protein 2	A4UGR9	5	5	2.5
X-ray repair cross-complementing protein 5	P13010	10	10	24.7
X-ray repair cross-complementing protein 6	P12956	3	3	8.9
Zinc-alpha-2-glycoprotein	P25311	14	14	54.4
Zyxin	Q15942	10	10	28
